# Trends in wildlife rehabilitation rescues and animal fate across a six-year period in New South Wales, Australia

**DOI:** 10.1371/journal.pone.0257209

**Published:** 2021-09-10

**Authors:** Alan B. C. Kwok, Ron Haering, Samantha K. Travers, Peter Stathis

**Affiliations:** 1 Independent consultant, Bensville, Australia; 2 New South Wales Department of Planning, New South Wales National Parks and Wildlife Service, Industry and Environment, Parramatta, Australia; 3 New South Wales Department of Planning, Industry and Environment, Parramatta, Australia; 4 Centre for Ecosystem Science, School of Biological, Earth and Environmental Sciences, University of New South Wales, Sydney, Australia; University of Sydney, AUSTRALIA

## Abstract

Globally, millions of animals are rescued and rehabilitated by wildlife carers each year. Information gathered in this process is useful for uncovering threats to native wildlife, particularly those from anthropogenic causes. However, few studies using rehabilitation data include a diverse range of fauna, cover large geographical areas, and consider long-term trends. Furthermore, few studies have statistically modelled causes of why animals come into care, and what are their chances of survival. This study draws on 469,553 rescues reported over six years by wildlife rehabilitators for 688 species of bird, reptile, and mammal from New South Wales, Australia. For birds and mammals, ‘abandoned/orphaned’ and ‘collisions with vehicles’ were the dominant causes for rescue, however for reptiles this was ‘unsuitable environment’. Overall rescue numbers were lowest in winter, and highest in spring, with six-times more ‘abandoned/orphaned’ individuals in spring than winter. Of the 364,461 rescues for which the fate of an animal was known, 92% fell within two categories: ‘dead’, ‘died or euthanased’ (54.8% of rescues with known fate) and animals that recovered and were subsequently released (37.1% of rescues with known fate). Modelling of the fate of animals indicated that the likelihood of animal survival (i.e. chance of: being released, left and observed, or permanent care), was related to the cause for rescue. In general, causes for rescue involving physical trauma (collisions, attacks, etc.) had a much lower likelihood of animals surviving than other causes such as ‘unsuitable environment’, ‘abandoned/orphaned’, and this also showed some dependence upon whether the animal was a bird, reptile, or mammal. This suggests rehabilitation efforts could be focused on particular threats or taxa to maximise success, depending on the desired outcomes. The results illustrate the sheer volume of work undertaken by rehabilitation volunteers and professionals toward both animal welfare and to the improvement of wildlife rehabilitation in the future.

## Introduction

Wildlife rescue and rehabilitation broadly involves the rescuing, treatment, and care of injured, sick or orphaned native animals. Ultimately the aim is to allow the animals to regain independence for release back into the wild or more suitable habitat [1,2]. When release is not an option early assessment to limit suffering through humane euthanasia is usually required [3]. Globally, the rescue and rehabilitation of native animals is primarily supported by the community through individual volunteers or as members of not-for-profit volunteer groups and wildlife rehabilitation centres. In the state of New South Wales in south-eastern Australia, the provision of wildlife rehabilitation services also relies heavily on volunteer participation and pro-bono services from private veterinary practices [4–6]. The sector is regulated under licence by the New South Wales Government (i.e. National Parks and Wildlife Service (NPWS) and within), totalling approximately 50 rehabilitation providers each year of various sizes and capacities. Roughly half of these are volunteer groups, with the remainder either rehabilitation facilities or independent rehabilitators. These providers are comprised of over 5,600 volunteers and operate across specified geographic areas over most of NSW. Home-based multi-species care is the sector’s primary mode of operation. These services are augmented by a small number of central facility-based organisations which are predominantly single species or similar species focused, and wildlife hospitals that also function as animal display establishments (i.e. zoos and aquaria; [7]).

Reporting protocols for wildlife rehabilitators vary depending on country, region, and organisation, but generally records are maintained for each individual animal rescued and rehabilitated. This valuable information generally includes the species of animal, location of rescue, why it came into care, physical condition including trauma sustained, and a range of other details (e.g. sex, life stage) [8–11].

Wildlife rehabilitation data have been used for a variety of purposes, predominantly elucidating threats to native animals and examining associated outcomes (e.g. [2,12–20]). Furthermore, studies have begun using these data to explore factors affecting release success, such as the life stage, condition, and sex of the animal (e.g. [2,9,11,21–23]). Single species studies are also common [15,24–28] including those for species with a threatened conservation status [2,13,29,30]). Wildlife rehabilitation data have also been used more broadly for disease surveillance [24,31–34], and in animal health science [22,35,36].

Despite a growing recognition of the utility of wildlife rehabilitation data, it remains a largely underutilised source of information. Wildlife rehabilitation likely generates millions of animal records per year globally [37], representing unique and information-rich datasets that may contain hundreds of species (e.g., [2,38,39]). While examining trends in causes for wildlife rescue is one of the more commonly researched topics, to date most studies primarily use descriptive statistics to examine these data, and there have been few studies that have attempted to statistically model rescue outcomes based on taxonomic group and the cause for rescue (though see [2,9]). Statistical models can capture levels of uncertainty around rescue outcomes and provide a more informative picture on the underlying factors and processes that contribute to rescue events and successful outcomes, or even allow predictions to be generated, leading to a greater understanding of the efficacy of the wildlife rehabilitation process and identification of areas for improvement.

In this study, we investigate patterns of wildlife rehabilitation across a six-year period from a broad geographical region in New South Wales using data collated from over 50 wildlife rehabilitation providers. New South Wales has about a third of all active wildlife rehabilitation volunteers in Australia and they annually respond to over 180,000 wildlife rescue assistance calls from the community [5,7,40]. To date few studies have modelled broad comprehensive trends across multiple animal taxa in wildlife rehabilitation, with most focusing on single species or a small groups of species (e.g. [29,41–43].

Here, we describe the general characteristics of wildlife rescues for birds, mammals, and reptiles in our study area, using both descriptive statistics and statistical models to answer three questions: (1) How do patterns of rescue change over time, both throughout the year and across the six-year period? (2) What are the main causes for rescue for each of the major taxonomic groups (birds, mammals, and reptiles)? (3) If inter-year temporal trends are minimal, what is the probability of survival for each of the animal groups, and how does this vary in relation to the cause for rescue?

## Methods

### Study area

Data from this study were obtained for the state of New South Wales (NSW), an area spanning over 800,000 km^2^ in south-eastern Australia. All wildlife rehabilitation providers are required to collect data about each animal rescued in a standard spreadsheet template as a condition of their licence [44] (S1 File). Reports are submitted annually to the NSW National Parks and Wildlife Service (NPWS) for each financial year (1st July to 30th June). The report includes data on taxonomic group and species, sex, and age class of each rescued animal as well as information about the rescue encounter (date, location, cause for rescue, animal condition) and the animal’s ultimate fate. For this study we extracted data from six consecutive years of reporting (1 July 2013 to 30 June 2018), submitted from 50 licensed wildlife rehabilitation providers.

The data presented in this study were acquired from volunteer wildlife rehabilitation providers acting in accordance with a licence issued under the NSW Biodiversity Conservation Act 2016. The licence permits these providers to undertake wildlife rehabilitation activities including harm (i.e. pursue, capture and/or euthanasia), possession, and release of protected animals in accordance with prescribed standards outlined in the NSW Department of Planning, Industry and Environment Codes of Practice [44] and/or where necessary by a licensed veterinarian under the NSW Veterinary Practices Act 2003. Data were obtained as part of licensing agreements with the NSW National Parks and Wildlife Service with written consent to publish approved by the NSW Department of Planning, Industry and Environment (DPIE). The work described in this study was not wildlife research requiring approval from an Animal Ethics Committee as it deals only with data.

### Data preparation

The initial reporting data contained 37 causes for rescue and 25 fates (S1 File). For the purpose of this study, rescues were pooled into a smaller number of categories (19 causes for rescue and 7 fates) (S2 File). Additionally, a binary score of animal fate (‘survived’ or ‘died’) was created for records where this was reported. ‘Survived’ includes animals that were left and observed at a rescue location, relocated to a more suitable habitat, released following care, or placed into permanent care. ‘Died’ includes animals that were found dead, died, or were euthanased.

In total, there were 872,087 records reported during the six-year (2013–14 to 2018–19) study period. Just over 97% of these came from three animal groups–birds, mammals, and reptiles. Of the total number of records, 402,534, (46%) were excluded from the descriptive analysis because they: a) did not contain any information about the animal, or the animal’s identification was ambiguous and could not be placed within a group (e.g. an ‘unidentified animal’); b) contained only sightings of animals and were not attended to in some way by a wildlife rehabilitator; c) were records of amphibians (373 records) or non-vertebrate fauna (e.g. spiders, insects, etc.); d) were non-avian marine vertebrates such as whales, seals, sharks, rays, fish etc; e) were reported as floating, drowned, or washed up animals (deemed an ambiguous cause for rescue, n = 48); f) contained both an ‘unknown’ cause for rescue and an ‘unknown’ fate; or f) were an introduced or spurious species (e.g. extinct, or out of known range). These exclusions resulted in a dataset for descriptive analysis of 469,553 records i.e. 54% of the initially reported amount.

For the descriptive analysis of causes for rescue, all records reported with an ‘unknown’ cause were excluded. Percentages therefore refer to proportions of known causes of rescue.

For statistical modelling of the likelihood of survival (see below), records with an ‘unknown’ cause for rescue were included. However, 107,604 records were excluded as they either reported an ambiguous fate (e.g. ‘unknown’ (50,664 records), ‘in care’ (54,428 records), ‘escaped from rescuer or carer’ (2,403 records), or were species that could not be confidently placed into animal subgroups (110 records). This resulted in a dataset of 361,949 records.

### Animal classification

Data were investigated at several scales of classification. Analyses were first conducted at the level of broad taxonomic group (class: bird, mammal, reptile). Subgroups were then created within each taxonomic group to allow for more detailed investigation of the fate of animals (S3 File). Subgroup classification was based on taxonomy, behaviour, and/or physical characteristics that could potentially reflect shared responses or reasons for coming into care. Where possible, we used classifications based on those that already exist in the wildlife rehabilitation literature (i.e. mammals, [19]; birds, [2]), however modifications and additions were made for the additional species in this study.

Seasonal trends were assessed using broad southern hemisphere seasons: Summer (December, January, February), Autumn (March, April, May), Winter (June, July, August), and Spring (September, October, November).

### Data analysis

To estimate the number of species for each survey year and for the entire survey period, species accumulation curves were created using the Chao1 and Jacknife2 indices in the PRIMER (Version 6) software [45].

We used binomial generalised linear mixed models (GLMMs) to assess the likelihood of survival (fate) for taxonomic groups in relation to causes for rescue. A binary classification of fate was used as a response variable for these models (survived: released, left and observed (represented as ‘1’ in the statistical models), or permanent care; or died: dead, died, or euthanased (‘0’)) to model the likelihood of a survival. Likelihood models were constructed using a negative binomial distribution within the *‘glmmadmb’* function from the ‘*glmmADMB’* package [46] within R (Version 4.0.3, R Core [47]. We ran two separate models, one for the fate of birds and mammals, and one for the fate of reptiles due to data limitations with cause for rescue for reptiles. The bird and mammal model was constructed to investigate the additive fixed effects of cause for rescue, the two animal groups (mammals and birds), plus their two-way interaction, with random intercepts for each group and subgroup. The reptile model contained only the fixed effect of 20 causes for rescue, and slope was allowed to vary for each of 8 subgroup levels. For each model, we estimated confidence intervals around the model parameters (fixed and random) using Wald Confidence intervals. In prior model versions, survey year was considered (2013–14 to 2018–19), however this did not improve model fit as there was little variation among years, therefore the most parsimonious model was selected and year was not included in final models.

## Results

### Reporting trends

During the six-year (2013–2014 to 2018–2019) survey period a total of 227 reports were submitted by wildlife rehabilitation providers to NPWS (Table 1). This represents on average, 37.8 ± 1.4 reports per year. This number is generally lower than the total number of active rehabilitation providers as some volunteer groups were unable to provide data reports each year due to internal governance or other technical reasons. The variation in the number of reports received each year were minor (Table 1).

**Table 1:** Number of reports submitted by licensed wildlife rehabilitation providers (groups, centres, and individual rehabilitators) from 2013–14 to 2018–19.

	Group and centres	Individuals	Total
**2013–14**	24	19	43
**2014–15**	29	11	40
**2015–16**	23	14	37
**2016–17**	23	11	34
**2017–18**	26	12	38
**2018–19**	26	9	35

### Overall number of species

In total, there were 469,553 rescues reported during the survey period. Just over half of these (53.4%) were birds, 34.1% mammals, and 12.5% reptiles (Table 2, S4 File). In total, 688 species were reported across all animal groups. Birds were the most species rich (65.6% of all species) followed by reptiles (19.5%,) and mammals (15%) (Table 2, S3 File).

**Table 2:** Number of rescues and species reported for each animal group during the study period.

	Number of rescues	% of total rescues	Number of species
**Birds**	250,688	53.4	452
**Mammals**	160,155	34.1	103
**Reptiles**	58,710	12.5	134
**Total**	469,553	100%	689

**Fig 1:**
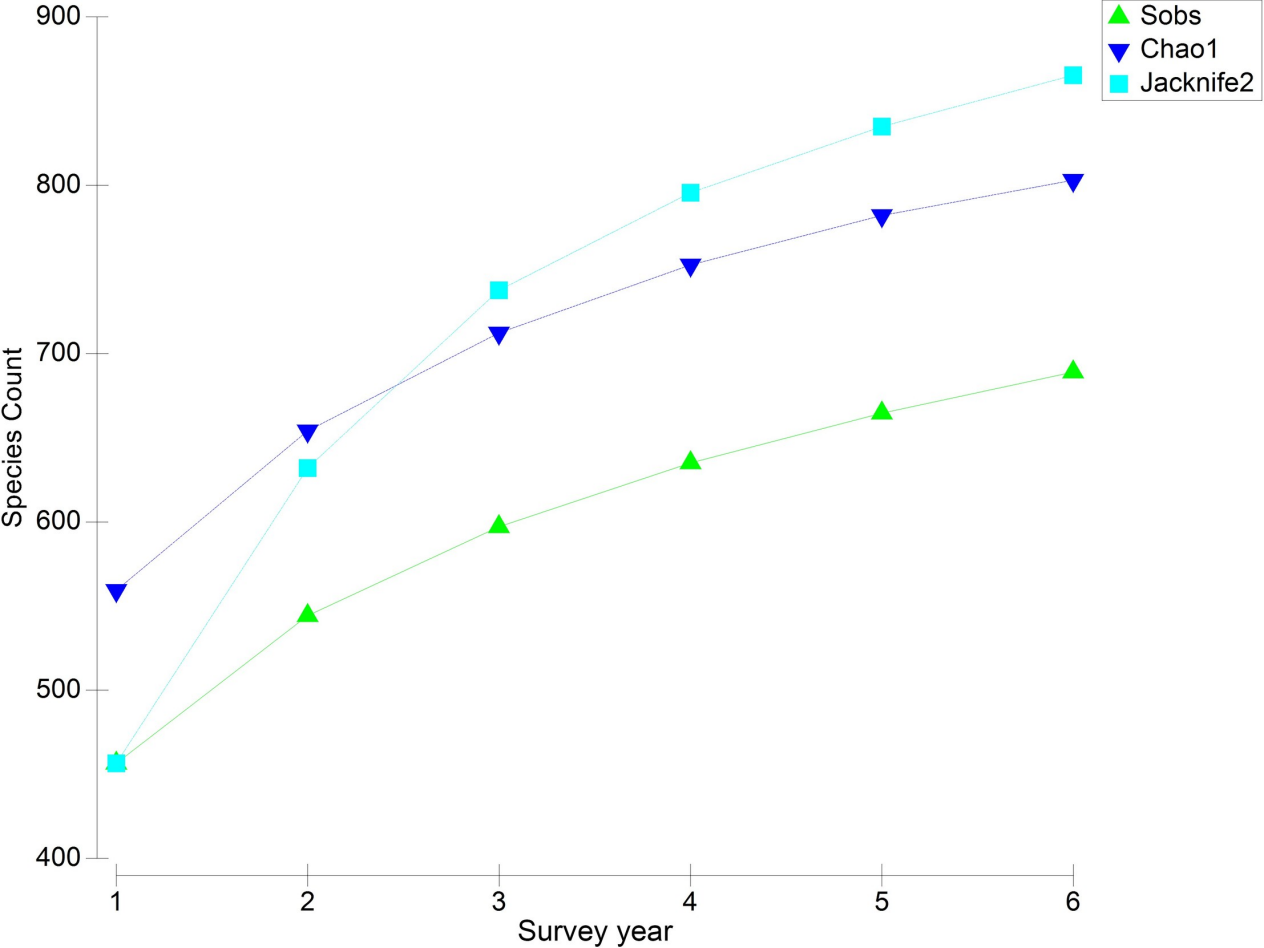
Species accumulation curve for all animal species, showing the rate of new species records accumulating across survey years. Survey year represents the year during the study period (i.e. 1 = 2013–14; 6 = 2018–19).

On average, there were 457 ± 7 species reported in total each year during the survey period (Table 3). The number of species reported from each animal group was stable from year to year, with no clear increases or decreases (Table 3). Although new species are reported for all taxonomic groups each year, the rate of new species records accumulating across years is slow and approaching a plateau (Fig 1). This pattern was consistent when each taxonomic group was analysed separately (S5 File).

**Table 3:** Number of species reported for each animal group each year during the study period.

		Birds	Mammals	Reptiles	Total
2013–14	No. rescues	38,856	24,295	8842	71,993
	No. species	301	69	69	439
2014–15	No. rescues	39,415	23,358	9601	72,374
	No. species	309	78	82	469
2015–16	No. rescues	40,489	23,401	10,375	74,265
	No. species	303	79	77	459
2016–17	No. rescues	41,354	28,899	10,232	80,485
	No. species	299	72	75	446
2017–18	No. rescues	41,971	28,890	8373	79,234
	No. species	317	78	87	482
2018–19	No. rescues	48,603	31,312	11,287	91,202
	No. species	291	73	80	444
	**Total rescues**	**250,688**	**160,155**	**58,710**	**469,553**

### Threatened species

Overall, 147 species with a conservation status listed as Threatened under the *NSW Biodiversity Conservation Act 2016* (BC Act) were reported across the six-year survey period, representing 21.3% of the total number of species rescued. Of these, 106 (72.1%), were classified as ‘Vulnerable’, 26 (17.7%) were ‘Endangered’, and 15 (10.2%) ‘Critically Endangered’ (Table 4). Across all animal groups, 58.5% of threatened species were birds, 31.3% were mammals, and 10.2% were reptiles (Table 5). The relative proportion of species classified as Threatened in the BC Act within each taxonomic group was variable. Approximately 44.7% of mammal species, 19% of bird species, and 11.2% of reptile species were classified as Threatened (Table 5). Of all individual animals rescued that were a threatened species, 92.2% were mammals, followed by 7.3% birds, and <1% reptiles (Table 5).

**Table 4:** Number of New South Wales threatened species reported for each animal group.

	Vulnerable	Endangered	Critically endangered	Total
**Birds**	65	13	8	86
**Mammals**	30	9	7	46
**Reptiles**	11	4		15
**Total**	106	26	15	147

**Table 5:** Number of New South Wales threatened species per animal group, as a percent of total species in that group and of the total number of threatened species across all groups.

	Number of threatened species	% of number of total number species in group	% of total threatened species	Number of threatened species rescues
**Birds**	86	19.0	58.5	1573
**Mammals**	46	44.7	31.3	19,879
**Reptiles**	15	11.2	10.2	104

### Causes for rescue

Overall, 46% of all rescues were attributed to a specific cause, with the remainder being reported as ‘unknown’. Of the known causes for rescue, three were dominant across all taxonomic groups: ‘collisions with vehicles’ (24.3%), ‘abandoned/orphaned’ (20.1%), and ‘unsuitable environment’ (16.8%) (Table 6). Another three causes account for about a further 20% of rescues (7.1% ‘entangled/trapped’; 6.2% ‘collisions with other objects’; and 5.1% ‘diseased’ individuals), with thirteen other causes responsible for the remaining 20.4% of rescues (Table 6).

**Table 6:** Number of rescues and reported cause for rescue, and the contribution of each cause of admission to the total number of rescues. Values are ranked from highest contributor to lowest based on number of records.

	Number of records	Cumulative contribution to total number of rescues (%)
Collision with vehicle	52,727	24.3
Abandoned/Orphaned	43,653	44.4
Unsuitable environment	36,416	61.2
Entangled/Trapped	15,444	68.3
Collision with other object	13,433	74.5
Disease	11,057	79.6
Attacked by other	10,137	84.3
Attacked by dog	8581	88.2
Attacked by cat	8472	92.1
Nuisance	3862	93.9
Weather—Unspecified	3796	95.6
Weather—Drought/heat	1871	96.5
Electrocution	1609	97.3
Domestic/Captivity issue	1599	98.0
Weather—Storm	1531	98.7
Poisoned	1059	99.2
Suboptimal condition	736	99.5
Fouled by Substance	682	99.8
Weather—Fire	370	100.00

**Fig 2:**
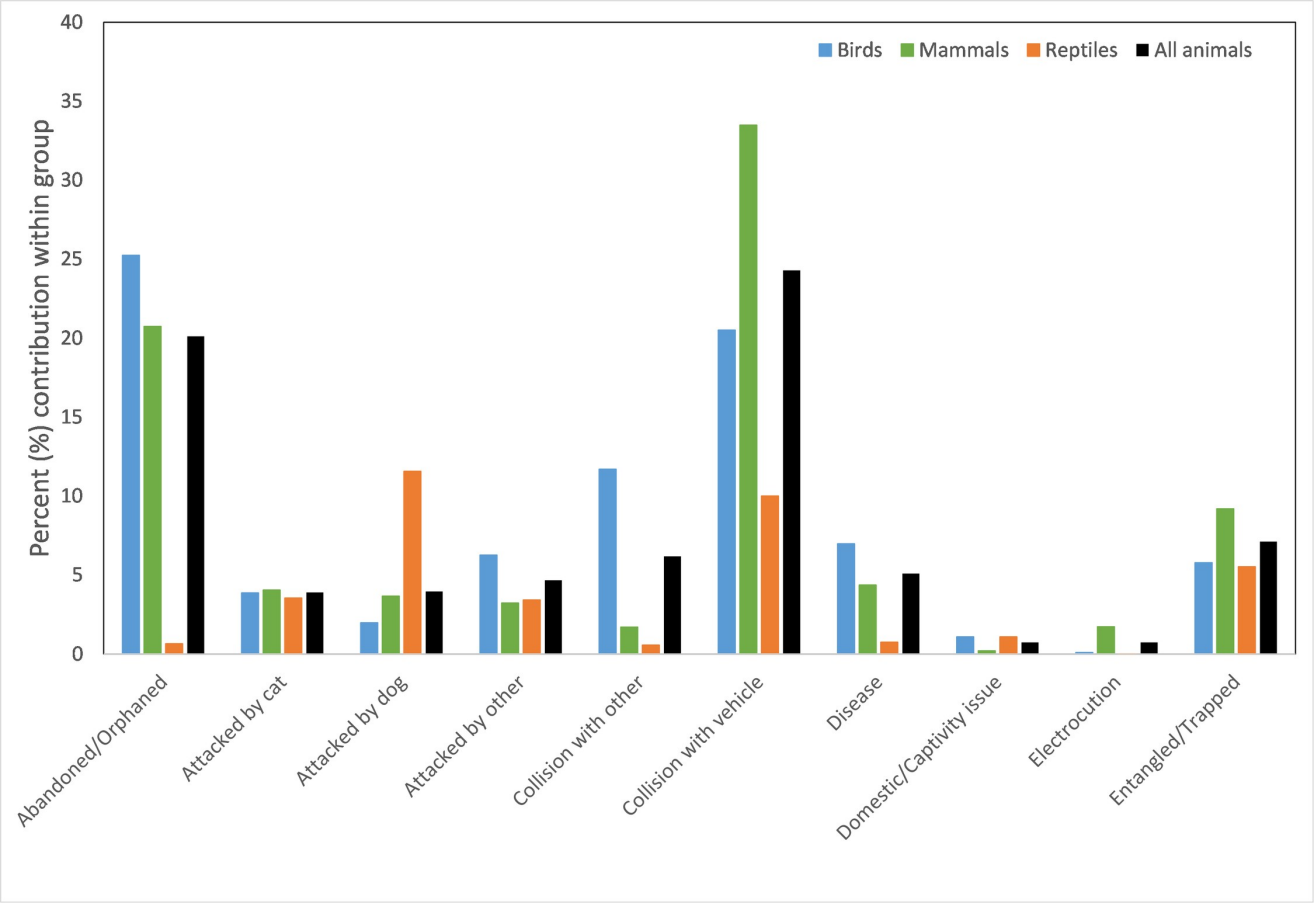
Cause for rescue (%) within each animal group, for top 10 highest ranked causes across all animals.

The number of rescues attributed to each cause varied between taxonomic groups (Table 7, Fig 2, S4 File). The two most common causes for bird and mammal rescues were ‘abandonment/orphaned’ (25.2% for birds and 20.8% for mammals) and ‘collisions with vehicles’, (20.5% for birds and 33.5% mammals). About 12% of birds were found injured from collisions with other objects such as windows and buildings and 12% rescued from an ‘unsuitable environment’ (mammals were about 11%). A further 9.2% of mammal rescues were animals ‘entangled/trapped’ in netting or wire.

**Table 7:** Number of records for each animal group and for each cause for rescue, and percent (%) of rescues within each group attributed to each cause for rescue.

	Birds		Mammals		Reptiles		Total
	No. rescues	% of rescues within group	No. rescues	% of rescues within group	No. rescues	% of rescues within group	No. rescues
**Abandoned/Orphaned**	25,411	25.2	18,049	20.7	193	0.7	43,653
**Attacked by cat**	3896	3.9	3534	4.1	1042	3.6	8472
**Attacked by dog**	1997	2.0	3189	3.7	3395	11.6	8581
**Attacked by other**	6316	6.3	2817	3.2	1004	3.4	10,137
**Collision with other**	11,783	11.7	1484	1.7	166	0.6	13,433
**Collision with vehicle**	20,651	20.5	29,139	33.5	2937	10.0	52,727
**Disease**	7036	7.0	3799	4.4	222	0.8	11,057
**Domestic/Captivity issue**	1102	1.1	177	0.2	320	1.1	1599
**Electrocution**	99	0.1	1507	1.7	3	0.01	1609
**Entangled/Trapped**	5820	5.8	8002	9.2	1622	5.5	15,444
**Nuisance**	379	0.4	899	1.0	2584	8.8	3862
**Poisoned**	583	0.6	437	0.5	39	0.1	1059
**Suboptimal condition**	168	0.2	531	0.6	37	0.1	736
**Weather—Drought/heat**	96	0.1	1772	2.0	3	0.01	1871
**Weather—Storm**	1372	1.4	146	0.2	13	0.04	1531
**Unsuitable environment**	11,531	11.5	9278	10.7	15,607	53.2	36,416
**Fouled by Substance**	482	0.5	143	0.2	57	0.2	682
**Weather—Fire**	36	0.04	316	0.4	18	0.1	370
**Weather—Unspecified**	1915	1.9	1816	2.1	65	0.2	3796
**Total**	100,673	100	87,035	100	29,327	100	217,035

For reptiles, ‘unsuitable environment’ was the most common cause (53.2% of rescues) followed by ‘attacks from dogs’ (11.6% of rescues) (Table 7). Reptiles were much more likely to be reported as ‘nuisance’ fauna (8.8%) than mammals (1%) or birds (0.4%) (Table 7).

Within each animal group, the cause for rescue did show some variation according to sub-group (S5 File). For example, for honeyeaters and passerines, abandoned/orphaned rescues comprised over 40% of the total number of rescues for that group, while this value was much lower for groups such as parrots and diurnal birds of prey (<12%).

### Trends across years

**Fig 3:**
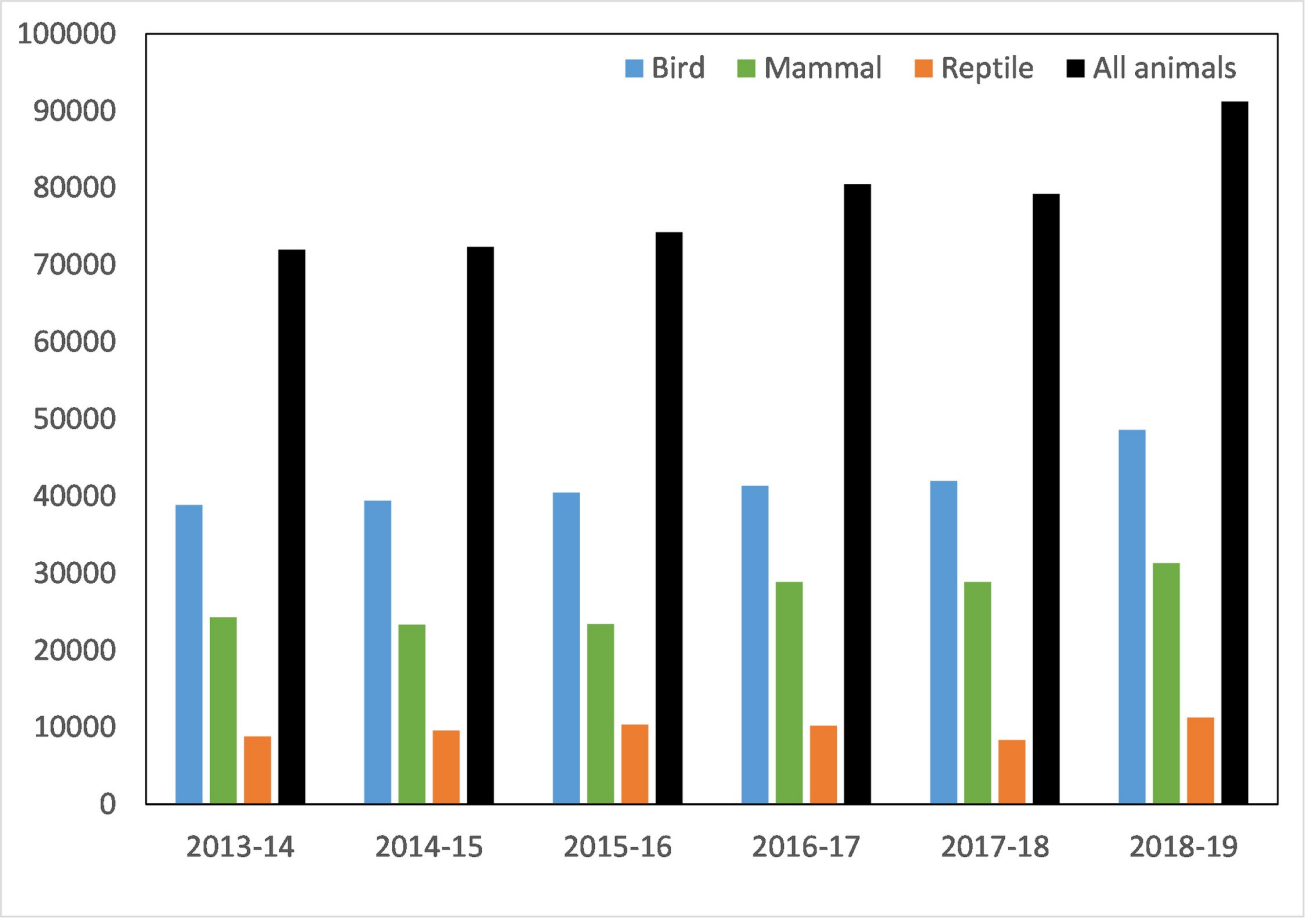
Number of rescue records per year for each animal group, and in total.

**Fig 4:**
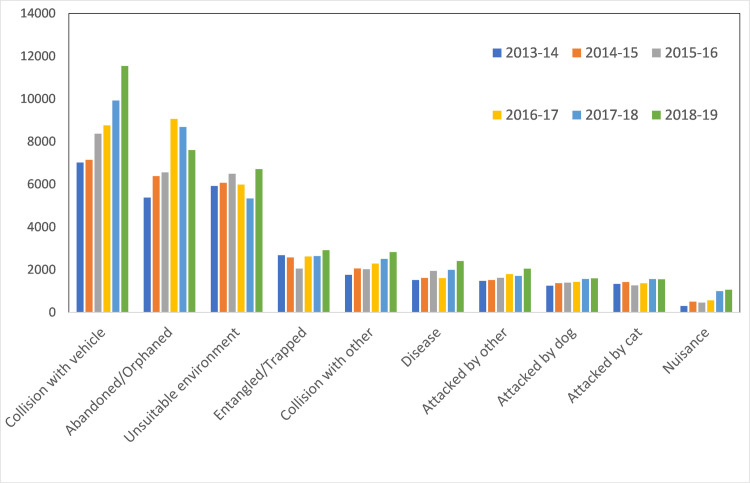
Cause for rescue by year for the 2013–14 to 2018–19 period, pooled for all animals and for top 10 highest ranked pooled causes.

Overall, average annual rescues for all animals combined (78,259 ± 2,962, mean ± SE) increased slightly during the study period, particularly from the last year of the survey (2018–19) (Table 3, Fig 3). The increase can be attributed to bird and mammal rescues with reptile rescues being mostly stable (Fig 3). Several causes for rescue appear to be increasing (in terms of the number of rescues) (Fig 4, S4 File). Most notable are collisions (both with vehicles, as well as other collisions) which have increased every year during the study period (Fig 4). Other causes for rescues such as ‘entangled’, ‘disease’ and ‘unsuitable environment’ showed slight increases, or inconsistent changes in the number of rescues (Fig 4, S4 File).

### Trends across months

**Fig 5:**
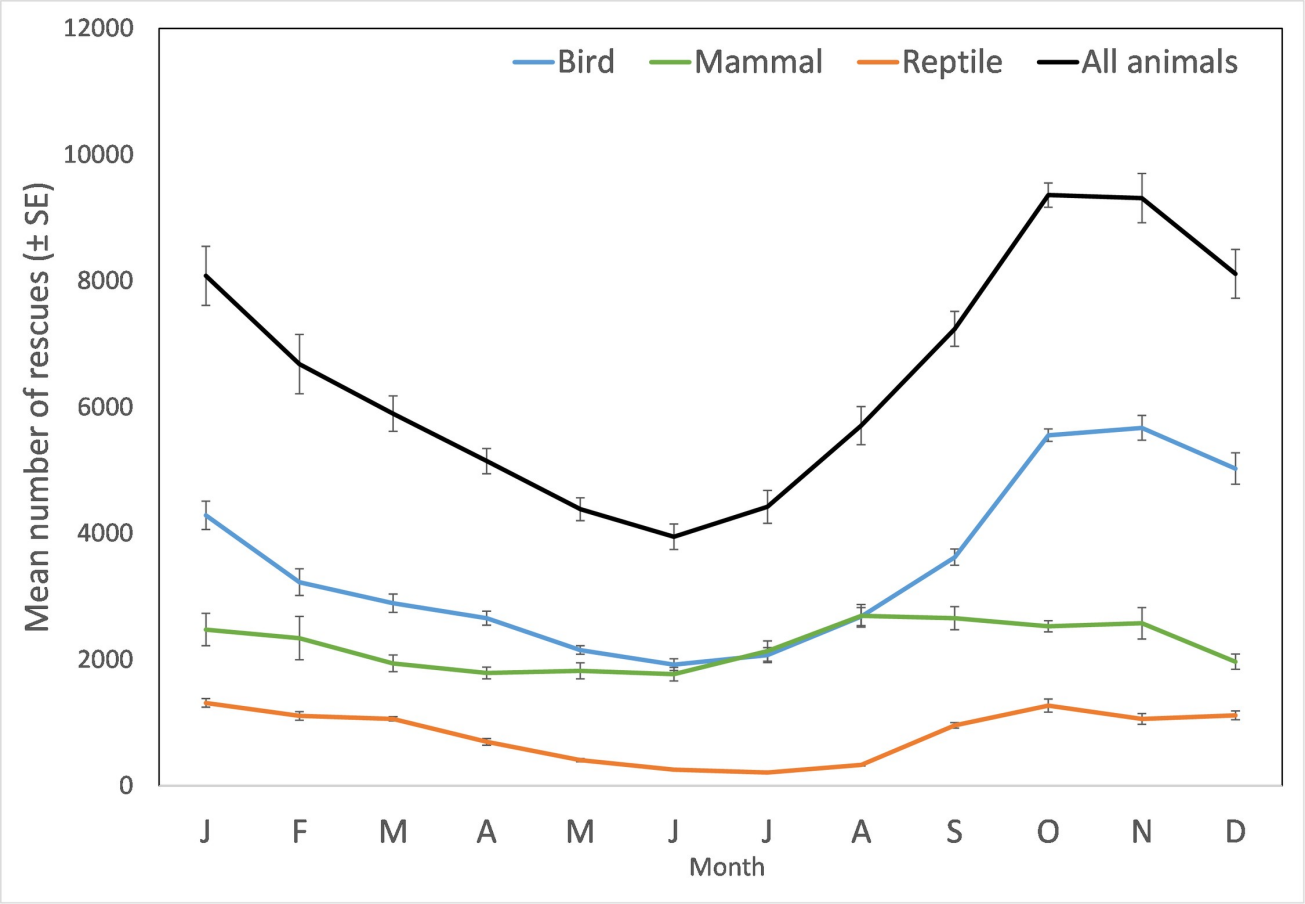
Mean ± SE number of rescues per month pooled for all years, for each animal group and all animals combined.

**Fig 6:**
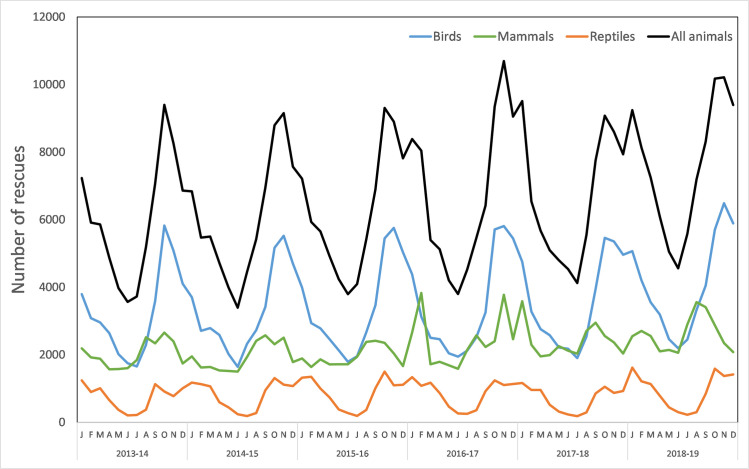
Number of records per year over the survey period, for birds, reptiles, mammals, and all animals.

Each month there were on average 6523 ± 232 rescues, however there is marked variation between months. The volume of rescues peak in October and November (spring), before steadily decreasing from December to January (early to mid-summer) to the lowest point in June (winter, Fig 5, S6 File). There are approximately 2.4 times more rescues in October and November (about 9300) than in June (about 4000 rescues/month) (Fig 5). This pattern is apparent for birds and reptiles. For mammals the peak (August) and low (April) both occur slightly earlier than for the other taxa (Fig 5). These patterns of monthly variation consistently occur for every year of the study period (Fig 6, S7 File).

**Fig 7:**
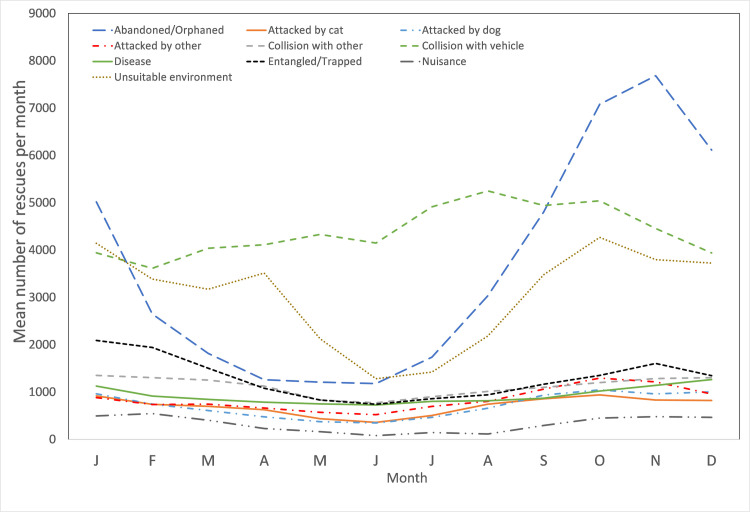
Mean number of rescues per month attributable to each of the top 8 pooled causes for rescue.

There was substantial monthly variation in the number of rescues for several causes for rescue (Fig 7, S8 File). For example, there are six times more ‘abandoned/orphaned’ rescues during the peak in November (1180 ± 65.4) than there are in June (197 ± 9), with a similar pattern in ‘unsuitable environment’ rescues. ‘Collisions with vehicles’, gradually increased from March to October, before declining for the following period (Fig 7).

**Fig 8:**
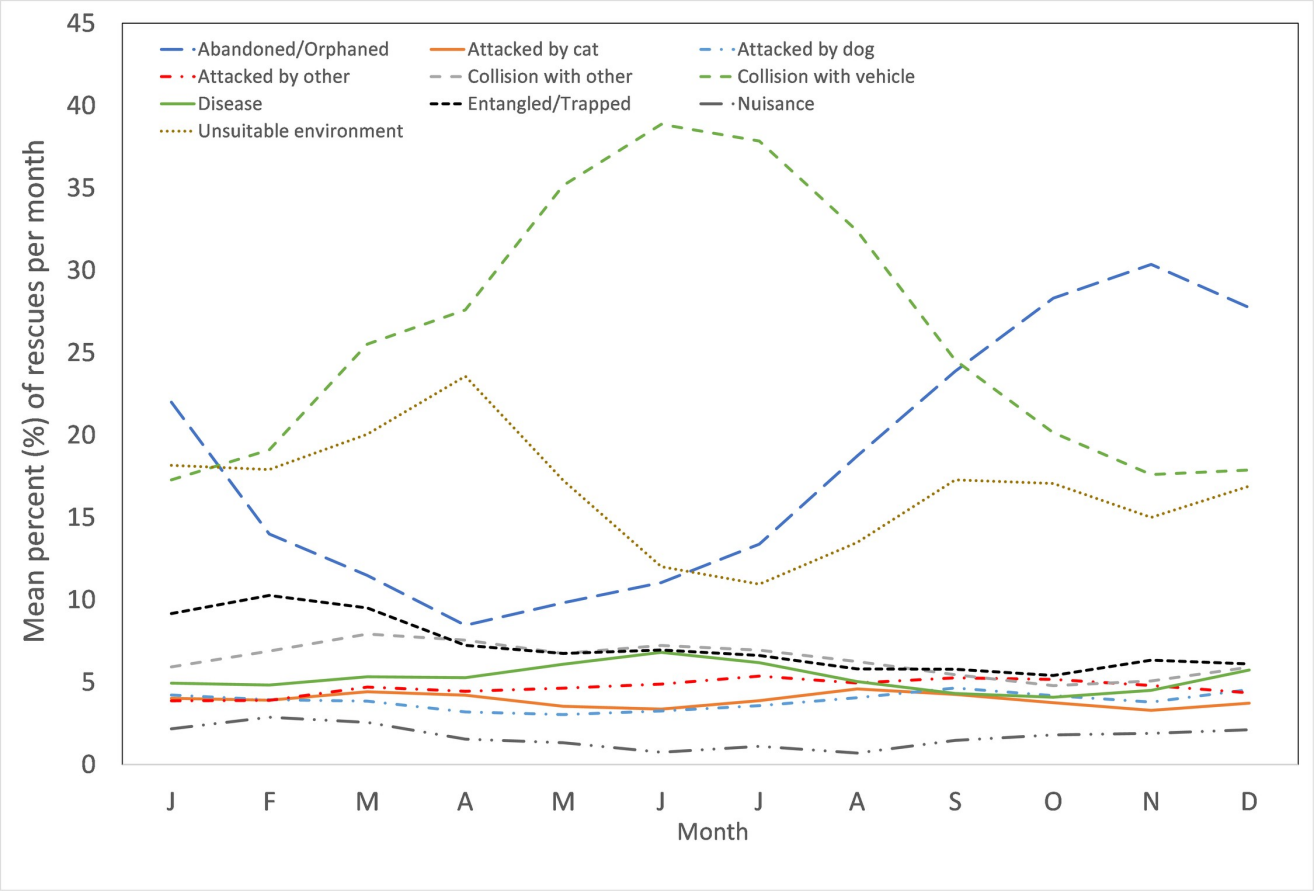
Percent (%) of rescues attributable to each of top 8 pooled causes for rescues within each month.

Some causes for rescue also showed monthly variation in the percentage contribution to the total number of rescues (Fig 8). The most noticeable change is an increase in the percentage of rescues due to ‘collisions with vehicles’ from January to June, with a mirrored decline from July onward. This pattern is in direct contrast to the percentage attributed to the ‘abandoned/orphaned’ cause for rescue. The remaining causes display only minor variations throughout the year.

### Fate and likelihood of survival

Of the 364,461 rescues for which a fate was known about 92% fell within two categories: Those that were dead, died or euthanased (54.8% of all rescues) and animals that recovered and were subsequently released (37.1% of all rescues) (Table 8). A further 7.2% of animals were reported as left and observed following a rescue callout and the remaining <1% were in long term rehabilitation or permanent care.

**Table 8:** Fate of animals for each taxonomic group, shown as the number of rescues and the percent of rescues within group.

	Birds		Mammals		Reptiles		All animals	
	No. rescues	%	No. rescues	%	No. rescues	%	No. rescues	%
**Died**	108,046	56.1	80,445	64.6	11,294	23.9	199,785	54.8
**Escaped rescuer or from care**	1652	<1	623	<1	128	<1	2403	<1
**Left and observed**	10,034	5.2	7819	6.3	8413	17.8	26,266	7.2
**Permanent/long term care**	260	<1	302	<1	172	<1	734	<1
**Released**	72,636	37.7	35,328	28.4	27,309	57.7	135,273	37.1
**Total**	192,628	100	124,517	100	47,316	100	364,461	100

**Fig 9:**
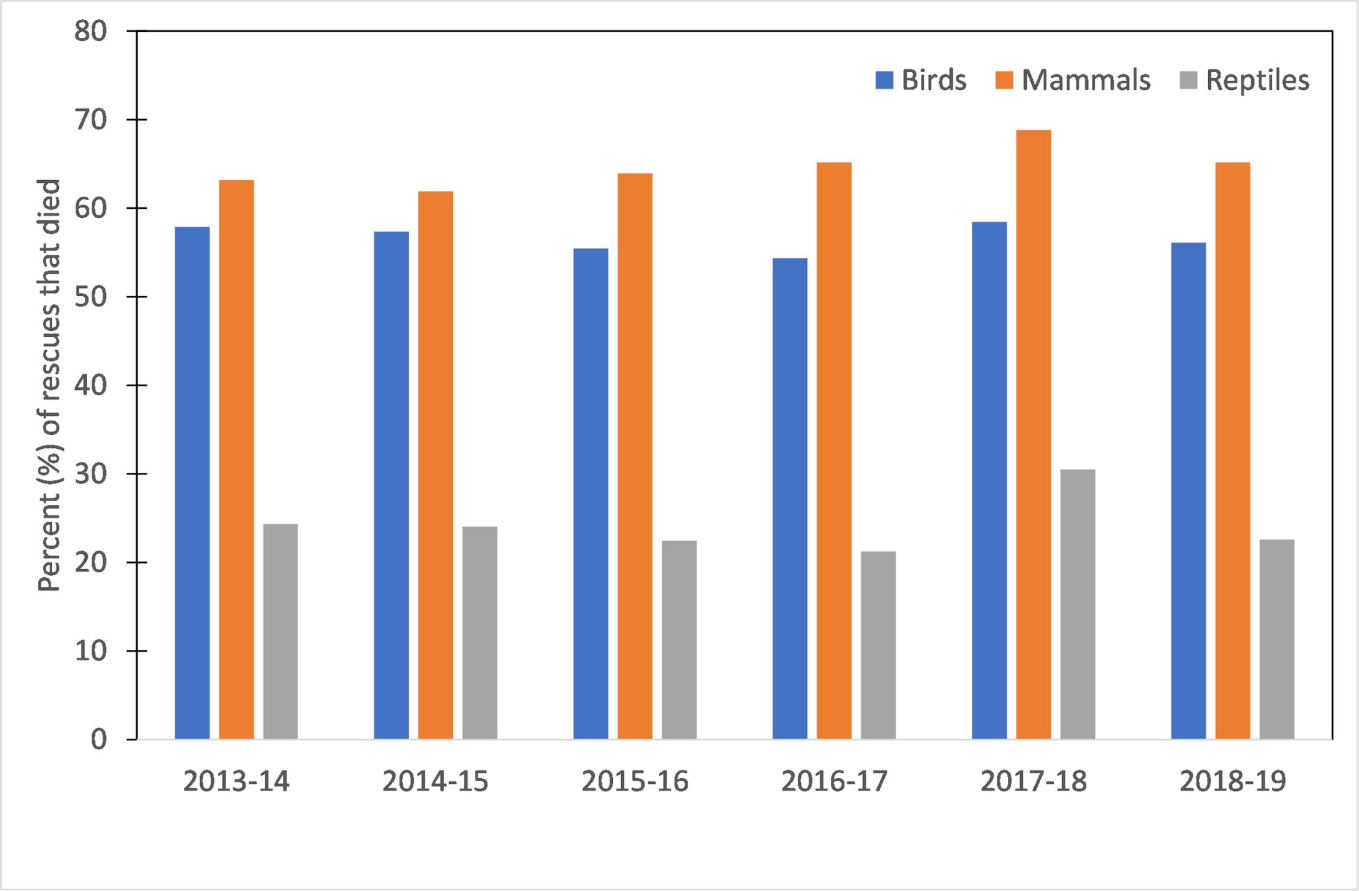
Percent (%) of rescues with a negative outcome for each group for each year, based on fate.

When the outcome of an animal’s fate was simplified into survived (released, left and observed, or permanent care) or died (dead, died, or euthanased), and with other outcomes omitted from analysis (i.e. escaped rescuer or from care), 55% of rescues (n = 199,785) survived, and 45% (n = 162,273) died. The proportion of rescues that survived to those that died was stable throughout the study period, though ‘died’ rescues in 2017–18 were slightly higher than other years (Table 9, S9 File). The outcome of a rescue was also dependent upon the taxonomic group (Fig 9). For example, the proportion of animals which had a negative outcome is substantially higher for mammals (65%) and birds (57%) than for reptiles (24%), across all years (Fig 9). The outcome of an animal’s fate also varied depending on the cause for rescue (Table 10), with marked differences between groups.

**Table 9:** Percent of rescues (%) classified as died or survived, based on fate.

	Died	Survived
**2013–14**	55.7	44.3
**2014–15**	54.7	45.3
**2015–16**	53.6	46.4
**2016–17**	54.1	45.9
**2017–18**	59.5	40.5
**2018–19**	55.1	44.9
**Overall**	55.5	44.5

**Table 10:** Fate (survived or died) for each cause for rescue and each animal group.

		Died	Survived	Total number of rescues
**Birds**	Abandoned/Orphaned	36.9%	63.1%	22,253
** **	Attacked by cat	77.3%	22.7%	3275
** **	Attacked by dog	70.1%	29.9%	1643
** **	Attacked by other	58.7%	41.3%	5291
** **	Collision with other	47.3%	52.7%	10,526
** **	Collision with vehicle	67.8%	32.2%	18,129
** **	Disease	89.3%	10.7%	6648
** **	Domestic/Captivity issue	34.3%	65.7%	402
** **	Electrocution	76.7%	23.3%	73
** **	Entangled/Trapped	32.4%	67.6%	5153
** **	Fouled by Substance	48.6%	51.4%	414
** **	Nuisance	6.1%	93.9%	343
** **	Poisoned	82.2%	17.8%	546
** **	Suboptimal condition	73%	27%	152
** **	Unknown	61.8%	38.2%	102,475
** **	Unsuitable environment	24.9%	75.1%	10,510
** **	Weather—Drought/heat	26.4%	73.6%	87
** **	Weather—Fire	57.1%	42.9%	28
** **	Weather—Storm	41.5%	58.5%	1281
** **	Weather—Unspecified	36.9%	63.1%	1747
**Mammals**	Abandoned/Orphaned	50.9%	49.1%	13,927
** **	Attacked by cat	73.9%	26.1%	3005
** **	Attacked by dog	73.5%	26.5%	2803
** **	Attacked by other	65.2%	34.8%	2401
** **	Collision with other	76.1%	23.9%	1325
** **	Collision with vehicle	87.8%	12.2%	25,273
** **	Disease	75.1%	24.9%	3406
** **	Domestic/Captivity issue	36%	64%	125
** **	Electrocution	90.3%	9.7%	1419
** **	Entangled/Trapped	56%	44%	6384
** **	Fouled by Substance	68.1%	31.9%	119
** **	Nuisance	9%	91%	818
** **	Poisoned	82.3%	17.7%	419
** **	Suboptimal condition	79%	21%	500
** **	Unknown	63.5%	36.5%	50,008
** **	Unsuitable environment	17.1%	82.9%	8224
** **	Weather—Drought/heat	91%	9%	1724
** **	Weather—Fire	62.4%	37.6%	237
** **	Weather—Storm	38.3%	61.7%	128
** **	Weather—Unspecified	63.1%	36.9%	1649
**Reptiles**	Abandoned/Orphaned	12.5%	87.5%	168
** **	Attacked by cat	50.2%	49.8%	809
** **	Attacked by dog	65.6%	34.4%	2803
** **	Attacked by other	50.8%	49.2%	844
** **	Collision with other	60.1%	39.9%	143
** **	Collision with vehicle	67.4%	32.6%	2496
** **	Disease	37.3%	62.7%	201
** **	Domestic/Captivity issue	29.9%	70.1%	154
** **	Electrocution	100%	0%	2
** **	Entangled/Trapped	13.4%	86.6%	1444
** **	Fouled by Substance	20.9%	79.1%	43
** **	Nuisance	3.5%	96.5%	2233
** **	Poisoned	65.7%	34.3%	35
** **	Suboptimal condition	26.5%	73.5%	34
** **	Unknown	28.7%	71.3%	20,910
** **	Unsuitable environment	2.5%	97.5%	14,786
** **	Weather—Drought/heat	33.3%	66.7%	3
** **	Weather—Fire	60%	40%	15
** **	Weather—Storm	16.7%	83.3%	12
** **	Weather—Unspecified	22.6%	77.4%	53
**Total**		**55.2%**	**44.8%**	362,058

### Predicted likelihood of survival

**Fig 10:**
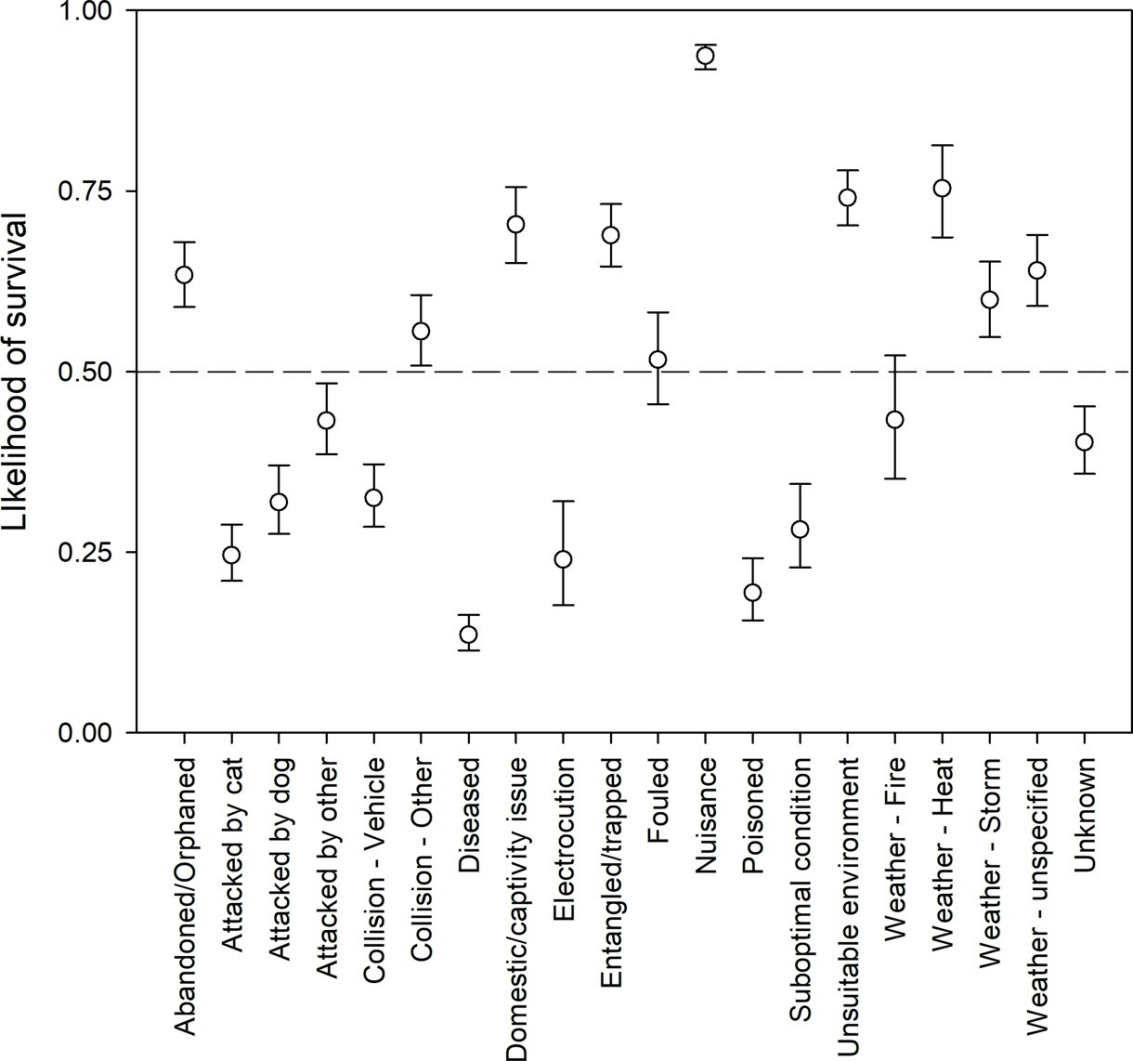
Likelihood of survival + 95% confidence intervals for birds and mammals when averaged, for each cause for rescue.

For birds and mammals combined, 10 causes for rescue generally resulted in a greater than 50% likelihood of survival outcomes (Fig 10). This was strongest for ‘domestic or captivity issues’, ‘nuisance’, and ‘unsuitable environment’.

**Fig 11:**
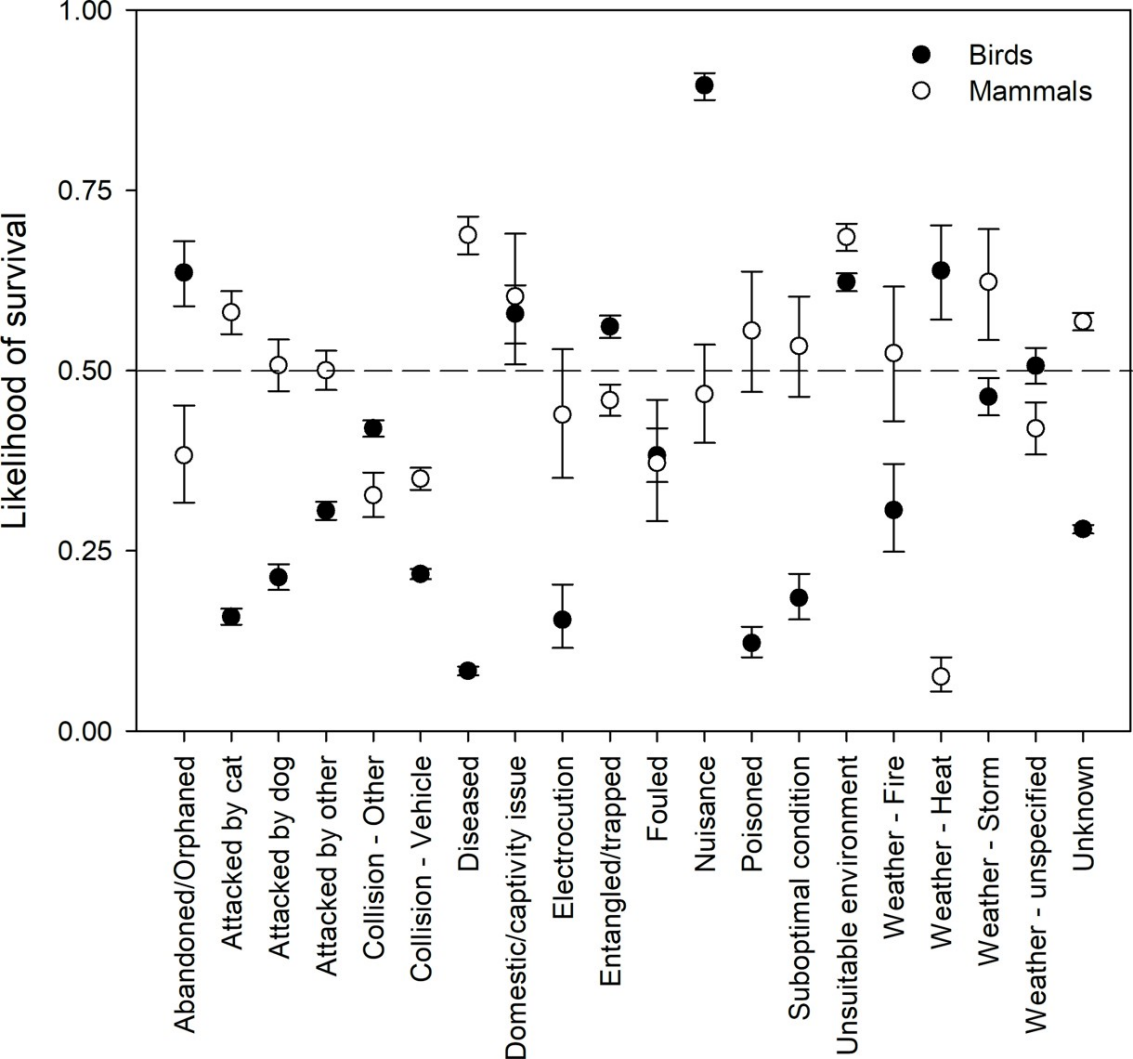
Likelihood of survival + 95% confidence intervals in relation to cause for rescue and animal group.

For birds and mammals combined, 10 of the 20 causes for rescue had less than a 50% likelihood of survival (Fig 10). For many causes for rescue, there was little variation in the probability of survival, particularly for attacks, collisions, and disease (Fig 10). The likelihood of survival did, however, show variation between taxonomic groups (Fig 11). In general, birds have a higher likelihood of survival compared to mammals, and for half of the causes for rescue (‘abandoned/orphaned, ‘attacked by dog’, ‘attacked by other’, ‘collisions with vehicles’, ‘collisions with other, ‘electrocution’, ‘entanglement/trapped’, ‘fouled’, ‘weather–heat’, and ‘weather–unspecified’) this difference was statistically significant. For collisions (with both vehicles, and other) and ‘weather–heat’ in particular, mammals had a significantly lower likelihood survival (Fig 11).

**Fig 12:**
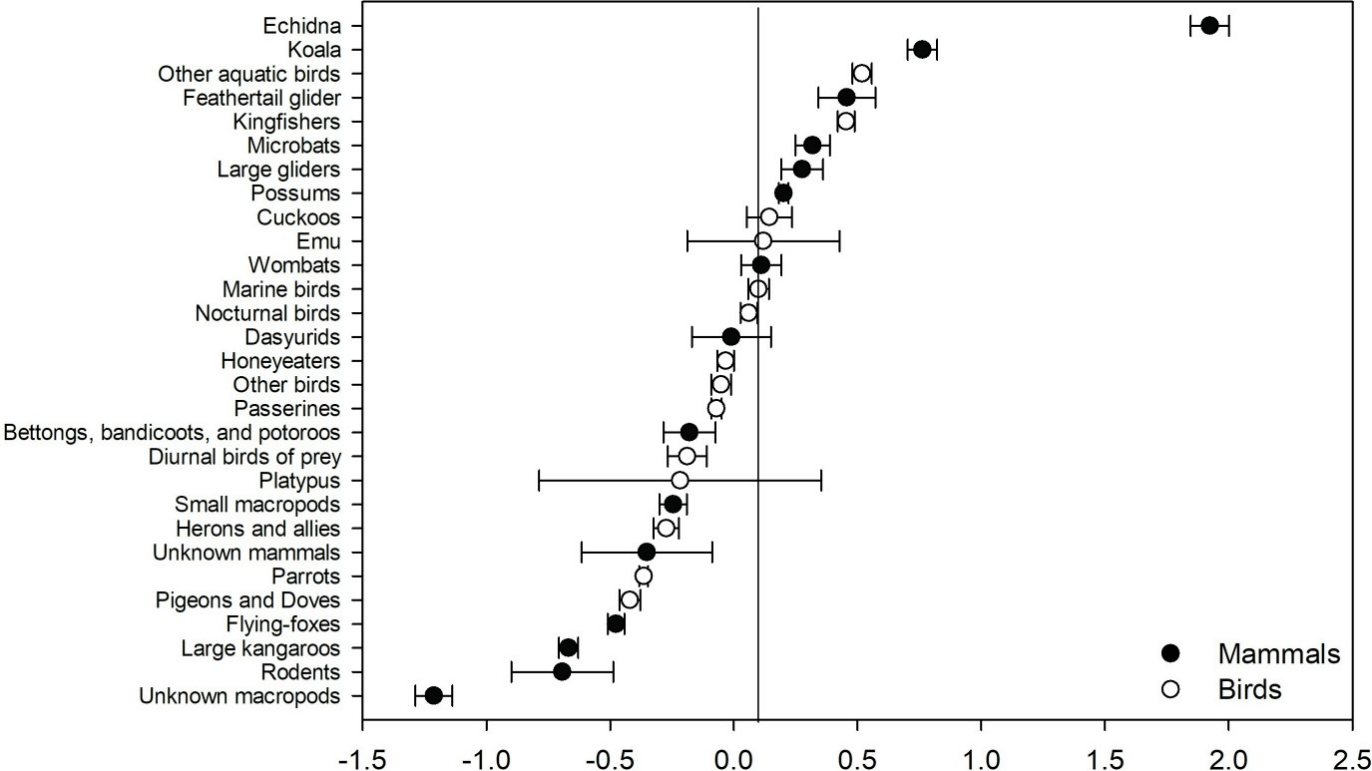
Response coefficients indicating the relative positive or negative response of bird and mammal subgroups amongst each other.

For ‘collisions–other’, ‘fouled’, and ‘weather–heat’, the likelihood of survival was greater than 0.5 for birds and lower than 0.5 for the mammals (Fig 11). In no cases did mammals have a significantly greater likelihood of survival than birds. ‘Nuisance’ rescues and those for animals reported in an unsuitable environment had a consistently high likelihood of survival for both birds and mammals (Fig 11). Predicted outcomes also indicate that the likelihood of survival varies between animal subgroups. For example, echidnas, and the koala generally fare much better than other subgroups such as rodents, and large kangaroos (Fig 12).

**Fig 13:**
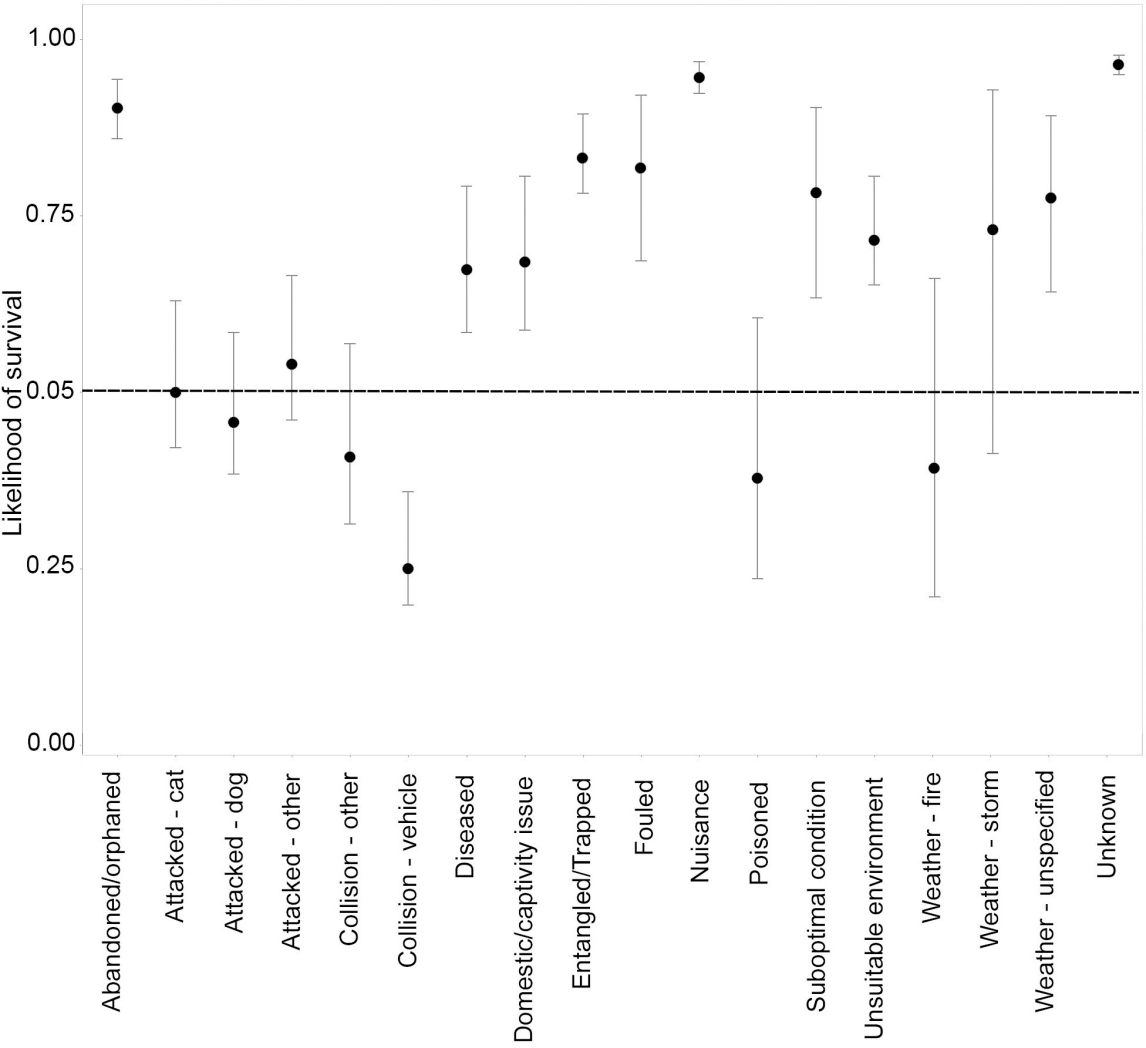
Likelihood of survival + 95% confidence intervals for reptiles in relation to cause for rescue.

**Fig 14:**
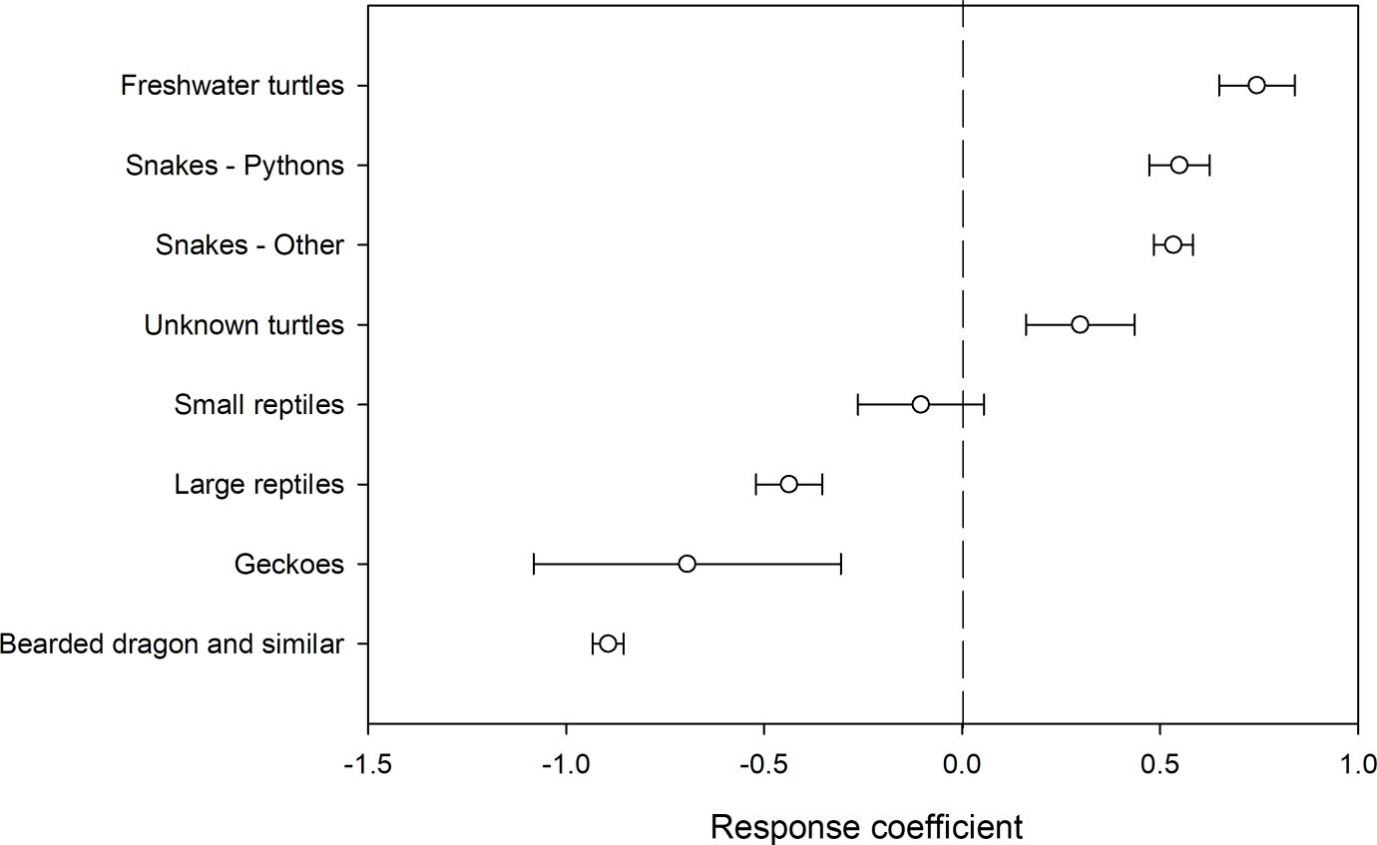
Response coefficient (+ - 95% CI) of reptile subgroups indicating their relative positive or negative response amongst each other.

For reptiles, the predicted likelihood of survival was generally greater or close to 50% for all causes for rescue, except ‘collisions with vehicle’, which was markedly lower (0.25) (Fig 13). There was, however, high variability in the fate of reptiles for most causes for rescue. As for birds and mammals, the subgroup of the animal influences a reptile’s likelihood of survival with snakes and turtles more likely to survive than other reptiles such as geckos, and bearded dragons (Fig 14).

## Discussion

This six-year study presents the largest single analysis of wildlife rehabilitation data in New South Wales and builds on a growing understanding of the direct factors contributing to the rescue of injured, sick and orphaned Australian native animals and their likelihood of survival. The results shed light on four key outcomes: (1) The relatively high annual number of animal rescues occurring over the study area with birds the most common and most species rich animal group represented; (2) abandonment or orphaning of individuals and collisions with vehicles are the dominant direct reasons why birds and mammals are reported and rescued, accounting for nearly half of all rescues, where a cause was attributed; (3) there is substantial seasonal variation in rescue volume throughout the year, but only minor increases across the study period; (4) for most causes for rescue, the modelled likelihood of survival for birds and mammals is <0.5, but substantially higher for reptiles (>0.6). Additionally, variation in predicted outcomes is low for birds and mammals but is much higher for reptiles. Despite clear trends at the broadest taxonomic level, our data indicate that likelihood of survival also depends on both the group of the animal and the subgroup to which it belongs.

### Volume and diversity of animals rescued

We found wildlife rehabilitation providers in New South Wales rescued on average about 78,260 native animals each year between 2013–19. The number of rescues appears have risen slightly over the study period, but this may be a function of improved reporting and an increase in volunteer numbers collecting data [48]. The annual average is a 41% increase on the 45,000 animals reported by the last study that collated rescue numbers in New South Wales across all animal groups [49]. In our study, a relatively high proportion of rescues were of birds (53.4% of all rescues), and similar results have been reported previously for rehabilitators [49] and in private veterinary hospitals [6,50].

Most species rescued were common and widespread, though threatened species represent a substantial number of species and rescues in certain cases. Threatened animals accounted for only 4.6% of the total number of animals rescued but more than a fifth of all species in the study. Nearly half (45%) of all mammal species rescued were threatened species, and the majority of threatened animal rescues were of mammals (92%). The latter figure is due to high number of rescues of two threatened species: the grey-headed flying fox *Pteropus poliocephalus* [42] and koala *Phascolarctos cinereus* [48,51]. The high richness of threatened species rescues in New South Wales is previously unreported, and these data may help inform conservation management programs for these species [37]. The true number of threatened species rescued is also likely to be higher when marine species are taken into consideration [41,48,51].

Seasonal variation in wildlife rescue volume has been well reported in Australia [1,19] and other countries [27,28,39,52,53], with more rescues occurring during Spring-Summer than Autumn-Winter. This generally reflects increased levels of breeding and rearing of young (particularly for birds), as well as greater movement and activity of animals during these periods. In Australia, Spring-Summer can bring periods of extreme heat and rainfall associated flooding events, which can also result in fauna requiring rescue and rehabilitation (e.g. flying-foxes, [42]). Birds showed the most distinct seasonal pattern which correlates with the high influx of abandoned/orphaned birds rescued during that time. In contrast, for some groups of animals the incidences of rescue can be higher during the colder months. For example, our data indicate that rescues for large macropods are greater in the cooler June-August period, particularly for collisions with vehicles. This aligns with a range of other studies [54,55], including motor vehicle insurance data [56]. Higher macropod collisions during cooler months is thought to be due to the spatial availability of food (lower growth of food in pastures, and greater growth of food along moister roadside verges during these periods), as well as more night-time driving hours and therefore greater potential contact with nocturnal animals [55]. These patterns interact with higher mortality for certain causes for rescue, resulting in a lower likelihood of survival for animals such as large macropods (see below).

### Causes for rescue

Our study has shown that in New South Wales the two dominant causes reported for bird and mammal rescues are ‘abandoned/orphaned’, and ‘collisions with vehicles’. Together, these two causes account for about 45% of all known reasons for rescue. ‘Abandoned/orphaned’ animals are often cited as a key cause for rescue in Australia [1,19,49] and other countries [2,10,18,20,27,57–59], often involving healthy juvenile animals with a relatively high likelihood of recovery and future release. High incident rates of wildlife ‘collisions with vehicles’ show some geographic variation but are particularly prevalent in Australia and overseas in studies with increasing road networks and vehicular traffic [19,29,38–40], and result in relatively high rates of mortality due to the traumatic nature of injuries sustained by affected animals.

Just over 60% of all reptile rescues were assigned as ‘unsuitable environment’ or ‘nuisance’ in this study. In these cases, the animal is not necessarily injured, but the member of the public either perceives them to be in danger (e.g. confined in a yard with a dog or cat), or more likely that they do not want the animal close to their dwelling. This was particularly the case for snakes, which represented most of the ‘nuisance’ records across all animals. This result is consistent with previous studies that found nearly 40% of lizards and over 70% of snakes were rescued in Sydney, New South Wales because residents wanted them removed from their property [43]. Studies investigating reptiles within wildlife rehabilitation are limited, and those published to date have not included a category alluding to ‘unsuitable environment’ or ‘nuisance’ [53,60,61]. This is because animal receiving centres in these studies were mostly wildlife veterinary hospitals treating reptiles affected by trauma related injuries from vehicles and dog and cat attacks. The inclusion of this category of cause for rescue is important for monitoring human-wildlife conflicts which clearly can be animal specific.

Wildlife rehabilitation data are often used to show the direct threats to native animals, particularly those related to anthropogenic activities [1,19,57,58]. Within this context it is important to recognise that threats to the welfare of animals are varied and often there may be more than one reason why an animal needs rescue [8,13]. An animal may be abandoned or orphaned because its parent has been attacked by a dog or has been in a collision with a vehicle. This is particularly the case for marsupials (e.g. kangaroos, possums) as they carry pouch young, but is also relevant for birds which suffer a higher number of attacks from other animals. Underlying these direct threats are also landscape scale impacts from fragmentation and clearing of habitat [18,19] that may be difficult to detect by rehabilitators on ground. For example, increases in wild bird collisions in Spain have been shown to be due to increased road infrastructure and vehicle activity [39]. Similarly, urban landscape change has been shown to negatively affect body condition and increase the prevalence of Chlamydia disease in koalas in south-east Queensland [62], and such change would not be reported in rehabilitation data. In general, as is the case with this dataset, a large proportion of wildlife rescue and rehabilitation occurs in urban and peri-urban environments. It is in these environments where both humans are most densely populated, and where the most traumatic threats to wildlife are likely to occur (e.g. collisions).

### Predicted likelihood of survival

For many rescued animals, the chances of survival are low. Our study shows that across all animal groups, more than half died or were humanely euthanased, while about 37% were released back to the wild. The release rate is slightly higher than the 31% reported for New South Wales approximately twenty years ago [49]. The overall mortality rate of about 55% is similar to that found in Queensland, Australia [19] and in studies elsewhere [2,57]. Anthropogenic driven causes such as all collision, attacks, and entangled/trapped have previously been shown to be associated with high mortality outcomes, and for some mammal species this may be as high as 90% [19,48,51]. In contrast, the majority of reptiles are healthy animals in ‘unsuitable environments’ or are a ‘nuisance’, and subsequently had release rates of greater than 75%. Importantly, in our study we were able to utilise a large dataset to create robust statistical models that predict the likelihood of survival for rescued animals. These analyses indicate that not only did many causes for rescue have a low predicted likelihood of survival, but the variation around these predictions was low.

In addition to trends evident for broad animal groups, variability in survivorship is also likely to occur within animal group and for different species. This is related to cause for rescue, the nature of injures, condition at admission [9,11], as well the sex, and age of the animal (e.g. [9,17,42,63]. Individual species may have particular traits, behaviours, breeding and movement patterns that may expose them to particular threats [19], and these may also be age-specific (e.g. [43]. Future analysis at finer taxonomic or functional levels will allow us to gain a better understanding of the cause of rescue events and will assist in the development of strategies both for threat mitigation, and for treatment once in care. This may be particularly the case for threatened and/or migratory species, for which specific conservation actions are often enacted (e.g. [51]).

### Implications for the volunteer wildlife rehabilitation sector

This study demonstrates the significant effort volunteers make to the rescue of injured, sick and orphaned native animals in New South Wales. They are frontline responders often working in challenging environments at significant personal cost and stress [5,7,40]. In New South Wales alone, the total value of volunteer services each year was estimated to be in excess of $27 million AUD and for private veterinary hospitals about $1.1 million AUD [5,6]. There are implications for the welfare of animals and their prospects for recovery if they are not adequately assessed and rehabilitated, and consequently there has been a significant investment in the development of minimum standards in the wildlife rehabilitation sector in NSW through the development of Codes of Practice [6]. These standards are being augmented with the training standards to ensure volunteers across the sector are competent to implement the requirements of each Code of Practice [4]. These standards need to be complemented by a program of mentoring [5,64] and ongoing volunteer training opportunities such as webinars and conferences that connect volunteers to other veterinary and conservation networks. It is clear from this study that such standards and training are necessary given the volume of rescues being undertaken each year.

This study also highlights the benefits of the State’s fauna authority working collaboratively with the volunteer wildlife rehabilitation sector to standardise data collection processes and report periodically so it can be systematically collated and reported on in a holistic fashion [1,19,58]. There are limitations and challenges when utilising wildlife rehabilitation data [7,9,37,58], including addressing poor species identification and a high proportion of unknown causes for rescue or animal fates. However, this does not negate the potential usefulness of the data so long as these caveats are considered are addressed carefully. Furthermore, it is crucial to demonstrate to the wildlife rehabilitators the potential uses of the data, particularly as to how their reporting is used to inform landscape scale processes such as environmental planning, research and threatened species management [37]. This is also important in creating an understanding amongst rehabilitators as to the importance of accurate data collection and reporting, which is generally regarded as a low priority by some providers. Additionally, further collaboration should be sought between scientists, rehabilitators, and veterinarians to investigate post-release success of animals on wild populations [37].

### Conclusion

From our study, it is clear that there are discernible trends in wildlife rehabilitation in NSW. Birds, mammals, and reptiles each are subject to specific threats, each with their own probability of a successful rehabilitation outcome. In general, causes for rescue such as collisions or animal attacks that involve some sort of physical trauma result in relatively poor chances for a successful outcome. In contrast, less physically traumatic, such as those for abandoned or orphaned individuals, or where humans simply do not want the animals in their presence, have a much relatively high likelihood of a successful outcome. Detailed statistical analyses indicate that most causes for rescue have very little variation in their likelihood of survival for an animal, suggesting that these data could be used to better inform triage of animals in the future, whilst also allowing focus on those threats for which there are greater chances of success both for individuals and populations.

## Supporting information

S1 File Reporting template for wildlife rehabilitation providers. (XLS)

S2 File Classifications of originally reported “encounter type” into “cause for rescue” for analyses in this study. (DOCX)

S3 File List of species and classification of species into sub-groups for statistical analyses. (DOCX)

S4 File Number of rescues—cause for rescue by animal group and year. (XLSX)

S5 File Species accumulation curves for birds, mammals, and reptiles. (DOCX)

S6 File Number of rescues, and percentage attributed to cause for rescue for each subgroup (where cause for rescue was reported). (XLSX)

S7 File Number of rescues by animal group, sub-group, year, and encounter month. (XLSX)

S8 File Cause for rescue by animal group and month. (XLSX)

S9 File Fate (positive/negative) records as a percentage of rescues, by animal group and year. (XLSX)
