# Supplementary material for: Trends in wildlife rehabilitation rescues and animal fate across a six-year period in New South Wales, Australia
Source: PLoS One. 2021 Sep 10;16(9):e0257209. doi: 10.1371/journal.pone.0257209 (PMC8432793; doi:10.1371/journal.pone.0257209)
Supplement: S2 File — (DOCX) [file pone.0257209.s002.docx]

S2 File: Classifications of originally reported “encounter type” into “cause for rescue” for analyses in this study

| **Original reporting classification** | **Classification for analyses** |
| --- | --- |
| Abandoned/Orphaned | Abandoned/Orphaned |
| Attack - Bird | Attacked by other |
| Attack - Cat | Attacked by cat |
| Attack - Dog | Attacked by dog |
| Attack - Fox | Attacked by other |
| Attack - Same Species | Attacked by other |
| Attack - Suspected | Attacked by other |
| Collision - Motor Vehicle | Collision with vehicle |
| Collision - Other | Collision with other |
| Dependent On Parent Taken Into Care | Abandoned/Orphaned |
| Disease - Chlamydia | Disease |
| Disease - External parasite | Disease |
| Disease - Internal parasite | Disease |
| Disease - Mange | Disease |
| Disease - Other | Disease |
| Disease - Runner Syndrome | Disease |
| Distress In Captivity | Domestic/Captivity issue |
| Domestic Escape/Release | Domestic/Captivity issue |
| Electrocution | Electrocution |
| Entanglement - fishing tackle | Entangled/Trapped |
| Entanglement - netting/wire | Entangled/Trapped |
| Fighting in Wild | Attacked by other |
| Fouled By Substance | Fouled by Substance |
| Geriatric | Suboptimal condition |
| Habitat Loss | Unknown |
| Moulting | Suboptimal condition |
| Nuisance/Problem Fauna | Nuisance |
| Poisoned | Poisoned |
| Trapped | Entangled/Trapped |
| Unclassified/Other | Unknown |
| Unknown | Unknown |
| Unsuitable Environment | Unsuitable environment |
| Unwanted Pet | Domestic/Captivity issue |
| Weather Condition - Drought | Weather - Drought/heat |
| Weather Condition - Fire | Weather - Fire |
| Weather Condition - Flood | Weather - Storm |
| Weather Condition - Storm | Weather - Storm |
