# Supplementary material for: Trends in wildlife rehabilitation rescues and animal fate across a six-year period in New South Wales, Australia
Source: PLoS One. 2021 Sep 10;16(9):e0257209. doi: 10.1371/journal.pone.0257209 (PMC8432793; doi:10.1371/journal.pone.0257209)
Supplement: S3 File — (DOCX) [file pone.0257209.s003.docx]

**S2 File: List of species and classification of species into sub-groups for statistical analyses**

| **Group** | **Sub-group** | **Common name** | **Scientific name** |
| --- | --- | --- | --- |
| Aves | Cuckoos | Fan-Tailed Cuckoo | *Cacomantis flabelliformis* |
|  |  | Pallid Cuckoo | *Cacomantis pallidus* |
|  |  | Brush Cuckoo | *Cacomantis variolosus* |
|  |  | Pheasant Coucal | *Centropus phasianinus* |
|  |  | Horsfield's Bronze-Cuckoo | *Chalcites basalis* |
|  |  | Shining Bronze-Cuckoo | *Chalcites lucidus* |
|  |  | Little Bronze-Cuckoo | *Chalcites minutillus* |
|  |  | Black-Eared Cuckoo | *Chalcites osculans* |
|  |  | Oriental Cuckoo | *Cuculus optatus* |
|  |  | Eastern Koel | *Eudynamys orientalis* |
|  |  | Asian Koel | *Eudynamys scolopaceus* |
|  |  | Channel-Billed Cuckoo | *Scythrops novaehollandiae* |
|  | Diurnal birds of prey | Collared Sparrowhawk | *Accipiter cirrocephalus* |
|  |  | Brown Goshawk | *Accipiter fasciatus* |
|  |  | Grey Goshawk | *Accipiter novaehollandiae* |
|  |  | Wedge-Tailed Eagle | *Aquila audax* |
|  |  | Pacific Baza | *Aviceda subcristata* |
|  |  | Swamp Harrier | *Circus approximans* |
|  |  | Spotted Harrier | *Circus assimilis* |
|  |  | Black-Shouldered Kite | *Elanus axillaris* |
|  |  | Red Goshawk | *Erythrotriorchis radiatus* |
|  |  | Brown Falcon | *Falco berigora* |
|  |  | Nankeen Kestrel | *Falco cenchroides* |
|  |  | Australian Hobby | *Falco longipennis* |
|  |  | Peregrine Falcon | *Falco peregrinus* |
|  |  | Black Falcon | *Falco subniger* |
|  |  | White-Bellied Sea-Eagle | *Haliaeetus leucogaster* |
|  |  | Brahminy Kite | *Haliastur indus* |
|  |  | Whistling Kite | *Haliastur sphenurus* |
|  |  | Little Eagle | *Hieraaetus morphnoides* |
|  |  | Square-Tailed Kite | *Lophoictinia isura* |
|  |  | Black Kite | *Milvus migrans* |
|  |  | Osprey | *Pandion haliaetus* |
|  | Emu | Emu | *Dromaius novaehollandiae* |
|  | Herons and allies | Darter | *Anhinga melanogaster* |
|  |  | Australasian Darter | *Anhinga novaehollandiae* |
|  |  | Great Egret | *Ardea alba* |
|  |  | Grey Heron | *Ardea cinerea* |
|  |  | Cattle Egret | *Ardea ibis* |
|  |  | Intermediate Egret | *Ardea intermedia* |
|  |  | Eastern Great Egret | *Ardea modesta* |
|  |  | White-Necked Heron | *Ardea pacifica* |
|  |  | Australasian Bittern | *Botaurus poiciloptilus* |
|  |  | Striated Heron | *Butorides striatus* |
|  |  | Little Egret | *Egretta garzetta* |
|  |  | White-Faced Heron | *Egretta novaehollandiae* |
|  |  | Eastern Reef Egret | *Egretta sacra* |
|  |  | Black-Necked Stork | *Ephippiorhynchus asiaticus* |
|  |  | Great Frigatebird | *Fregata minor* |
|  |  | Black Bittern | *Ixobrychus flavicollis* |
|  |  | Little Bittern | *Ixobrychus minutus* |
|  |  | Little Pied Cormorant | *Microcarbo melanoleucos* |
|  |  | Australasian Gannet | *Morus serrator* |
|  |  | Nankeen Night Heron | *Nycticorax caledonicus* |
|  |  | Abbott's Booby | *Papasula abbotti* |
|  |  | Australian Pelican | *Pelecanus conspicillatus* |
|  |  | Great Cormorant | *Phalacrocorax carbo* |
|  |  | Black-Faced Cormorant | *Phalacrocorax fuscescens* |
|  |  | Little Black Cormorant | *Phalacrocorax sulcirostris* |
|  |  | Pied Cormorant | *Phalacrocorax varius* |
|  |  | Yellow-Billed Spoonbill | *Platalea flavipes* |
|  |  | Royal Spoonbill | *Platalea regia* |
|  |  | Glossy Ibis | *Plegadis falcinellus* |
|  |  | Masked Booby | *Sula dactylatra* |
|  |  | Brown Booby | *Sula leucogaster* |
|  |  | Red-Footed Booby | *Sula sula* |
|  |  | Australian White Ibis | *Threskiornis molucca* |
|  |  | Straw-Necked Ibis | *Threskiornis spinicollis* |
|  | Kingfishers | Azure Kingfisher | *Ceyx azureus* |
|  |  | Little Kingfisher | *Ceyx pusilla* |
|  |  | Laughing Kookaburra | *Dacelo novaeguineae* |
|  |  | Collared Kingfisher | *Todiramphus chloris* |
|  |  | Forest Kingfisher | *Todiramphus macleayii* |
|  |  | Red-Backed Kingfisher | *Todiramphus pyrrhopygius* |
|  |  | Sacred Kingfisher | *Todiramphus sanctus* |
|  | Marine birds | Common Sandpiper | *Actitis hypoleucos* |
|  |  | Black Noddy | *Anous minutus* |
|  |  | Common Noddy | *Anous stolidus* |
|  |  | Flesh-Footed Shearwater | *Ardenna carneipes* |
|  |  | Sooty Shearwater | *Ardenna grisea* |
|  |  | Wedge-Tailed Shearwater | *Ardenna pacificus* |
|  |  | Short-Tailed Shearwater | *Ardenna tenuirostris* |
|  |  | Ruddy Turnstone | *Arenaria interpres* |
|  |  | Bush Stone-Curlew | *Burhinus grallarius* |
|  |  | Sharp-Tailed Sandpiper | *Calidris acuminata* |
|  |  | Curlew Sandpiper | *Calidris ferruginea* |
|  |  | White-Rumped Sandpiper | *Calidris fuscicollis* |
|  |  | Red-Necked Stint | *Calidris ruficollis* |
|  |  | Long-Toed Stint | *Calidris subminuta* |
|  |  | Streaked Shearwater | *Calonectris leucomelas* |
|  |  | Kentish Plover | *Charadrius alexandrinus* |
|  |  | Double-Banded Plover | *Charadrius bicinctus* |
|  |  | Greater Sand-Plover | *Charadrius leschenaultii* |
|  |  | Lesser Sand-Plover | *Charadrius mongolus* |
|  |  | Red-Capped Plover | *Charadrius ruficapillus* |
|  |  | Oriental Plover | *Charadrius veredus* |
|  |  | Silver Gull | *Chroicocephalus novaehollandiae* |
|  |  | Banded Stilt | *Cladorhynchus leucocephalus* |
|  |  | Wandering Albatross | *Diomedea exulans* |
|  |  | Black-Fronted Dotterel | *Elseyornis melanops* |
|  |  | Red-Kneed Dotterel | *Erythrogonys cinctus* |
|  |  | Beach Stone-Curlew | *Esacus magnirostris* |
|  |  | White-Bellied Storm-Petrel | *Fregetta grallaria* |
|  |  | Latham's Snipe | *Gallinago hardwickii* |
|  |  | Gull-Billed Tern | *Gelochelidon nilotica* |
|  |  | White Tern | *Gygis alba* |
|  |  | Sooty Oystercatcher | *Haematopus fuliginosus* |
|  |  | Pied Oystercatcher | *Haematopus longirostris* |
|  |  | Blue Petrel | *Halobaena caerulea* |
|  |  | Black-Winged Stilt | *Himantopus himantopus* |
|  |  | Caspian Tern | *Hydroprogne caspia* |
|  |  | Kelp Gull | *Larus dominicanus* |
|  |  | Pacific Gull | *Larus pacificus* |
|  |  | Bar-Tailed Godwit | *Limosa lapponica* |
|  |  | Black-Tailed Godwit | *Limosa limosa* |
|  |  | Kerguelen Petrel | *Lugensa brevirostris* |
|  |  | Southern Giant Petrel | *Macronectes giganteus* |
|  |  | Northern Giant-Petrel | *Macronectes halli* |
|  |  | Eastern Curlew | *Numenius madagascariensis* |
|  |  | Little Curlew | *Numenius minutus* |
|  |  | Whimbrel | *Numenius phaeopus* |
|  |  | Wilson's Storm-Petrel | *Oceanites oceanicus* |
|  |  | Sooty Tern | *Onychoprion fuscata* |
|  |  | Slender-Billed Prion | *Pachyptila belcheri* |
|  |  | Antarctic Prion | *Pachyptila desolata* |
|  |  | Fairy Prion | *Pachyptila turtur* |
|  |  | Broad-Billed Prion | *Pachyptila vittata* |
|  |  | White-Faced Storm-Petrel | *Pelagodroma marina* |
|  |  | Common Diving-Petrel | *Pelecanoides urinatrix* |
|  |  | White-Tailed Tropicbird | *Phaethon lepturus* |
|  |  | Red-Tailed Tropicbird | *Phaethon rubricauda* |
|  |  | Sooty Albatross | *Phoebetria fusca* |
|  |  | Light-Mantled Sooty Albatross | *Phoebetria palpebrata* |
|  |  | Pacific Golden Plover | *Pluvialis fulva* |
|  |  | Grey Plover | *Pluvialis squatarola* |
|  |  | Grey Petrel | *Procellaria cinerea* |
|  |  | Black Petrel | *Procellaria parkinsoni* |
|  |  | Westland Petrel | *Procellaria westlandica* |
|  |  | Grey Ternlet | *Procelsterna cerulea* |
|  |  | Cook's Petrel | *Pterodroma cookii* |
|  |  | Mottled Petrel | *Pterodroma inexpectata* |
|  |  | White-Headed Petrel | *Pterodroma lessonii* |
|  |  | Gould's Petrel | *Pterodroma leucoptera* |
|  |  | Great-Winged Petrel | *Pterodroma macroptera* |
|  |  | Black-Winged Petrel | *Pterodroma nigripennis* |
|  |  | Providence Petrel | *Pterodroma solandri* |
|  |  | Little Shearwater | *Puffinus assimilis* |
|  |  | Fluttering Shearwater | *Puffinus gavia* |
|  |  | Hutton's Shearwater | *Puffinus huttoni* |
|  |  | Audubon's Shearwater | *Puffinus lherminieri* |
|  |  | Red-Necked Avocet | *Recurvirostra novaehollandiae* |
|  |  | Australian Painted Snipe | *Rostratula australis* |
|  |  | Long-Tailed Jaeger | *Stercorarius longicaudus* |
|  |  | South Polar Skua | *Stercorarius maccormicki* |
|  |  | Arctic Jaeger | *Stercorarius parasiticus* |
|  |  | Roseate Tern | *Sterna dougallii* |
|  |  | Common Tern | *Sterna hirundo* |
|  |  | Arctic Tern | *Sterna paradisaea* |
|  |  | White-Fronted Tern | *Sterna striata* |
|  |  | Antarctic Tern | *Sterna vittata* |
|  |  | Little Tern | *Sternula albifrons* |
|  |  | Fairy Tern | *Sternula nereis* |
|  |  | Buller's Albatross | *Thalassarche bulleri* |
|  |  | Shy Albatross | *Thalassarche cauta* |
|  |  | White-Capped Albatross | *Thalassarche cauta steadi* |
|  |  | Yellow-Nosed Albatross | *Thalassarche chlororhynchos* |
|  |  | Grey-Headed Albatross | *Thalassarche chrysostoma* |
|  |  | Black-Browed Albatross | *Thalassarche melanophris* |
|  |  | Lesser Crested Tern | *Thalasseus bengalensis* |
|  |  | Crested Tern | *Thalasseus bergii* |
|  |  | Hooded Plover | *Thinornis rubricollis* |
|  |  | Green Sandpiper | *Tringa ochropus* |
|  |  | Red-Backed Button-Quail | *Turnix maculosus* |
|  |  | Red-Chested Button-Quail | *Turnix pyrrhothorax* |
|  |  | Painted Button-Quail | *Turnix varius* |
|  |  | Little Button-Quail | *Turnix velox* |
|  |  | Masked Lapwing | *Vanellus miles* |
|  |  | Spur-Winged Plover | *Vanellus miles novaehollandiae* |
|  |  | Banded Lapwing | *Vanellus tricolor* |
|  |  | Terek Sandpiper | *Xenus cinereus* |
|  | Other aquatic birds | Bush-Hen | *Amaurornis moluccanus* |
|  |  | Chestnut Teal | *Anas castanea* |
|  |  | Grey Teal | *Anas gracilis* |
|  |  | Pacific Black Duck | *Anas superciliosa* |
|  |  | Magpie Goose | *Anseranas semipalmata* |
|  |  | Hardhead | *Aythya australis* |
|  |  | Musk Duck | *Biziura lobata* |
|  |  | Cape Barren Goose | *Cereopsis novaehollandiae* |
|  |  | Australian Wood Duck | *Chenonetta jubata* |
|  |  | Black Swan | *Cygnus atratus* |
|  |  | Wandering Whistling-Duck | *Dendrocygna arcuata* |
|  |  | Plumed Whistling-Duck | *Dendrocygna eytoni* |
|  |  | Eurasian Coot | *Fulica atra* |
|  |  | Dusky Moorhen | *Gallinula tenebrosa* |
|  |  | Buff-Banded Rail | *Gallirallus philippensis* |
|  |  | Brolga | *Grus rubicunda* |
|  |  | Lewin's Rail | *Lewinia pectoralis* |
|  |  | Pink-Eared Duck | *Malacorhynchus membranaceus* |
|  |  | Green Pygmy-Goose | *Nettapus pulchellus* |
|  |  | Great Crested Grebe | *Podiceps cristatus* |
|  |  | Hoary-Headed Grebe | *Poliocephalus poliocephalus* |
|  |  | Purple Swamphen | *Porphyrio porphyrio* |
|  |  | Australian Spotted Crake | *Porzana fluminea* |
|  |  | Baillon's Crake | *Porzana pusilla* |
|  |  | Spotless Crake | *Porzana tabuensis* |
|  |  | Freckled Duck | *Stictonetta naevosa* |
|  |  | Australasian Grebe | *Tachybaptus novaehollandiae* |
|  |  | Little Grebe | *Tachybaptus ruficollis* |
|  |  | Australian Shelduck | *Tadorna tadornoides* |
|  |  | Black-Tailed Native-Hen | *Tribonyx ventralis* |
|  | Other birds | Australian Brush-Turkey | *Alectura lathami* |
|  |  | Fork-Tailed Swift | *Apus pacificus* |
|  |  | Stubble Quail | *Coturnix pectoralis* |
|  |  | Brown Quail | *Coturnix ypsilophora* |
|  |  | Dollarbird | *Eurystomus orientalis* |
|  |  | King Quail | *Excalfactoria chinensis* |
|  |  | White-Throated Needletail | *Hirundapus caudacutus* |
|  |  | Malleefowl | *Leipoa ocellata* |
|  |  | Rainbow Bee-Eater | *Merops ornatus* |
|  | Owls, nightjars and frogmouths | Australian Owlet-Nightjar | *Aegotheles cristatus* |
|  |  | Large-Tailed Nightjar | *Caprimulgus macrurus* |
|  |  | Spotted Nightjar | *Eurostopodus argus* |
|  |  | White-Throated Nightjar | *Eurostopodus mystacalis* |
|  |  | Barking Owl | *Ninox connivens* |
|  |  | Southern Boobook | *Ninox novaeseelandiae* |
|  |  | Powerful Owl | *Ninox strenua* |
|  |  | Marbled Frogmouth | *Podargus ocellatus* |
|  |  | Tawny Frogmouth | *Podargus strigoides* |
|  |  | Barn Owl | *Tyto alba* |
|  |  | Eastern Barn Owl | *Tyto javanica* |
|  |  | Eastern Grass Owl | *Tyto longimembris* |
|  |  | Masked Owl | *Tyto novaehollandiae* |
|  |  | Sooty Owl | *Tyto tenebricosa* |
|  | Parrots | Australian King-Parrot | *Alisterus scapularis* |
|  |  | Red-Winged Parrot | *Aprosmictus erythropterus* |
|  |  | Australian Ringneck | *Barnardius zonarius* |
|  |  | Mallee Ringneck | *Barnardius zonarius barnardi* |
|  |  | Sulphur-Crested Cockatoo | *Cacatua galerita* |
|  |  | Western Corella | *Cacatua pastinator* |
|  |  | Little Corella | *Cacatua sanguinea* |
|  |  | Long-Billed Corella | *Cacatua tenuirostris* |
|  |  | Gang-Gang Cockatoo | *Callocephalon fimbriatum* |
|  |  | Red-Tailed Black-Cockatoo | *Calyptorhynchus banksii* |
|  |  | Yellow-Tailed Black-Cockatoo | *Calyptorhynchus funereus* |
|  |  | Glossy Black-Cockatoo | *Calyptorhynchus lathami* |
|  |  | Eclectus Parrot | *Eclectus roratus* |
|  |  | Galah | *Eolophus roseicapillus* |
|  |  | Musk Lorikeet | *Glossopsitta concinna* |
|  |  | Purple-Crowned Lorikeet | *Glossopsitta porphyrocephala* |
|  |  | Little Lorikeet | *Glossopsitta pusilla* |
|  |  | Swift Parrot | *Lathamus discolor* |
|  |  | Major Mitchell's Cockatoo | *Lophochroa leadbeateri* |
|  |  | Budgerigar | *Melopsittacus undulatus* |
|  |  | Blue-Winged Parrot | *Neophema chrysostoma* |
|  |  | Elegant Parrot | *Neophema elegans* |
|  |  | Turquoise Parrot | *Neophema pulchella* |
|  |  | Scarlet-Chested Parrot | *Neophema splendida* |
|  |  | Bourke's Parrot | *Neopsephotus bourkii* |
|  |  | Blue Bonnet | *Northiella haematogaster* |
|  |  | Cockatiel | *Nymphicus hollandicus* |
|  |  | Ground Parrot | *Pezoporus wallicus* |
|  |  | Pale-Headed Rosella | *Platycercus adscitus* |
|  |  | Green Rosella | *Platycercus caledonicus* |
|  |  | Crimson Rosella | *Platycercus elegans* |
|  |  | Yellow Rosella | *Platycercus elegans flaveolus* |
|  |  | Eastern Rosella | *Platycercus eximius* |
|  |  | Western Rosella | *Platycercus icterotis* |
|  |  | Princess Parrot | *Polytelis alexandrae* |
|  |  | Regent Parrot | *Polytelis anthopeplus* |
|  |  | Superb Parrot | *Polytelis swainsonii* |
|  |  | Hooded Parrot | *Psephotus dissimilis* |
|  |  | Red-Rumped Parrot | *Psephotus haematonotus* |
|  |  | Mulga Parrot | *Psephotus varius* |
|  |  | Scaly-Breasted Lorikeet | *Trichoglossus chlorolepidotus* |
|  |  | Rainbow Lorikeet | *Trichoglossus haematodus* |
|  |  | Red-Collared Lorikeet | *Trichoglossus haematodus rubritorquis* |
|  | Passerines | Spiny-Cheeked Honeyeater | *Acanthagenys rufogularis* |
|  |  | Yellow-Rumped Thornbill | *Acanthiza chrysorrhoa* |
|  |  | Striated Thornbill | *Acanthiza lineata* |
|  |  | Yellow Thornbill | *Acanthiza nana* |
|  |  | Brown Thornbill | *Acanthiza pusilla* |
|  |  | Buff-Rumped Thornbill | *Acanthiza reguloides* |
|  |  | Eastern Spinebill | *Acanthorhynchus tenuirostris* |
|  |  | Scrubtit | *Acanthornis magna* |
|  |  | Australian Reed-Warbler | *Acrocephalus australis* |
|  |  | Green Catbird | *Ailuroedus crassirostris* |
|  |  | Red Wattlebird | *Anthochaera carunculata* |
|  |  | Little Wattlebird | *Anthochaera chrysoptera* |
|  |  | Yellow Wattlebird | *Anthochaera paradoxa* |
|  |  | Regent Honeyeater | *Anthochaera phrygia* |
|  |  | Australian Pipit | *Anthus novaeseelandiae* |
|  |  | Black-Faced Woodswallow | *Artamus cinereus* |
|  |  | Dusky Woodswallow | *Artamus cyanopterus* |
|  |  | White-Breasted Woodswallow | *Artamus leucorynchus* |
|  |  | Little Woodswallow | *Artamus minor* |
|  |  | Masked Woodswallow | *Artamus personatus* |
|  |  | White-Browed Woodswallow | *Artamus superciliosus* |
|  |  | Rufous Scrub-Bird | *Atrichornis rufescens* |
|  |  | Yellow-Faced Honeyeater | *Caligavis chrysops* |
|  |  | White-Eared Monarch | *Carterornis leucotis* |
|  |  | Pied Honeyeater | *Certhionyx variegatus* |
|  |  | Speckled Warbler | *Chthonicola sagittata* |
|  |  | Brown Songlark | *Cincloramphus cruralis* |
|  |  | Rufous Songlark | *Cincloramphus mathewsi* |
|  |  | Spotted Quail-Thrush | *Cinclosoma punctatum* |
|  |  | Golden-Headed Cisticola | *Cisticola exilis* |
|  |  | White-Browed Treecreeper | *Climacteris affinis* |
|  |  | Red-Browed Treecreeper | *Climacteris erythrops* |
|  |  | Brown Treecreeper | *Climacteris picumnus* |
|  |  | Grey Shrike-Thrush | *Colluricincla harmonica* |
|  |  | Little Shrike-Thrush | *Colluricincla megarhyncha* |
|  |  | Barred Cuckoo-Shrike | *Coracina lineata* |
|  |  | Ground Cuckoo-Shrike | *Coracina maxima* |
|  |  | Black-Faced Cuckoo-Shrike | *Coracina novaehollandiae* |
|  |  | White-Bellied Cuckoo-Shrike | *Coracina papuensis* |
|  |  | Cicadabird | *Coracina tenuirostris* |
|  |  | White-Winged Chough | *Corcorax melanorhamphos* |
|  |  | White-Throated Treecreeper | *Cormobates leucophaea* |
|  |  | Little Crow | *Corvus bennetti* |
|  |  | Australian Raven | *Corvus coronoides* |
|  |  | Little Raven | *Corvus mellori* |
|  |  | Torresian Crow | *Corvus orru* |
|  |  | Forest Raven | *Corvus tasmanicus* |
|  |  | Pied Butcherbird | *Cracticus nigrogularis* |
|  |  | Black Butcherbird | *Cracticus quoyi* |
|  |  | Australian Magpie | *Cracticus tibicen* |
|  |  | Grey Butcherbird | *Cracticus torquatus* |
|  |  | Eastern Bristlebird | *Dasyornis brachypterus* |
|  |  | Mistletoebird | *Dicaeum hirundinaceum* |
|  |  | Spangled Drongo | *Dicrurus bracteatus* |
|  |  | Southern Scrub-Robin | *Drymodes brunneopygia* |
|  |  | Blue-Faced Honeyeater | *Entomyzon cyanotis* |
|  |  | Eastern Yellow Robin | *Eopsaltria australis* |
|  |  | Crimson Chat | *Epthianura tricolor* |
|  |  | Singing Honeyeater | *Gavicalis virescens* |
|  |  | Western Gerygone | *Gerygone fusca* |
|  |  | Brown Gerygone | *Gerygone mouki* |
|  |  | White-Throated Gerygone | *Gerygone olivacea* |
|  |  | Magpie-Lark | *Grallina cyanoleuca* |
|  |  | Welcome Swallow | *Hirundo neoxena* |
|  |  | Barn Swallow | *Hirundo rustica* |
|  |  | Varied Triller | *Lalage leucomela* |
|  |  | White-Winged Triller | *Lalage sueurii* |
|  |  | Yellow-Tufted Honeyeater | *Lichenostomus melanops* |
|  |  | Brown Honeyeater | *Lichmera indistincta* |
|  |  | Lovely Fairy-Wren | *Malurus amabilis* |
|  |  | Superb Fairy-Wren | *Malurus cyaneus* |
|  |  | Variegated Fairy-Wren | *Malurus lamberti* |
|  |  | Red-Backed Fairy-Wren | *Malurus melanocephalus* |
|  |  | Blue-Breasted Fairy-Wren | *Malurus pulcherrimus* |
|  |  | Splendid Fairy-Wren | *Malurus splendens* |
|  |  | Yellow-Throated Miner | *Manorina flavigula* |
|  |  | Noisy Miner | *Manorina melanocephala* |
|  |  | Bell Miner | *Manorina melanophrys* |
|  |  | Black-Eared Miner | *Manorina melanotis* |
|  |  | Little Grassbird | *Megalurus gramineus* |
|  |  | Hooded Robin | *Melanodryas cucullata* |
|  |  | Lewin's Honeyeater | *Meliphaga lewinii* |
|  |  | Black-Headed Honeyeater | *Melithreptus affinis* |
|  |  | Brown-Headed Honeyeater | *Melithreptus brevirostris* |
|  |  | Black-Chinned Honeyeater | *Melithreptus gularis* |
|  |  | White-Naped Honeyeater | *Melithreptus lunatus* |
|  |  | Albert's Lyrebird | *Menura alberti* |
|  |  | Superb Lyrebird | *Menura novaehollandiae* |
|  |  | Jacky Winter | *Microeca fascinans* |
|  |  | Horsfield's Bushlark | *Mirafra javanica* |
|  |  | Black-Faced Monarch | *Monarcha melanopsis* |
|  |  | Satin Flycatcher | *Myiagra cyanoleuca* |
|  |  | Restless Flycatcher | *Myiagra inquieta* |
|  |  | Leaden Flycatcher | *Myiagra rubecula* |
|  |  | Scarlet Honeyeater | *Myzomela sanguinolenta* |
|  |  | Star Finch | *Neochmia ruficauda* |
|  |  | Red-Browed Finch | *Neochmia temporalis* |
|  |  | Yellow-Throated Honeyeater | *Nesoptilotis flavicollis* |
|  |  | White-Eared Honeyeater | *Nesoptilotis leucotis* |
|  |  | Crested Bellbird | *Oreoica gutturalis* |
|  |  | Rockwarbler | *Origma solitaria* |
|  |  | Olive-Backed Oriole | *Oriolus sagittatus* |
|  |  | Logrunner | *Orthonyx temminckii* |
|  |  | Olive Whistler | *Pachycephala olivacea* |
|  |  | Golden Whistler | *Pachycephala pectoralis* |
|  |  | Rufous Whistler | *Pachycephala rufiventris* |
|  |  | Spotted Pardalote | *Pardalotus punctatus* |
|  |  | Red-Browed Pardalote | *Pardalotus rubricatus* |
|  |  | Striated Pardalote | *Pardalotus striatus* |
|  |  | Fairy Martin | *Petrochelidon ariel* |
|  |  | Tree Martin | *Petrochelidon nigricans* |
|  |  | Scarlet Robin | *Petroica boodang* |
|  |  | Red-Capped Robin | *Petroica goodenovii* |
|  |  | Flame Robin | *Petroica phoenicea* |
|  |  | Pink Robin | *Petroica rodinogaster* |
|  |  | Rose Robin | *Petroica rosea* |
|  |  | Helmeted Friarbird | *Philemon buceroides* |
|  |  | Little Friarbird | *Philemon citreogularis* |
|  |  | Noisy Friarbird | *Philemon corniculatus* |
|  |  | White-Cheeked Honeyeater | *Phylidonyris niger* |
|  |  | New Holland Honeyeater | *Phylidonyris novaehollandiae* |
|  |  | Crescent Honeyeater | *Phylidonyris pyrrhoptera* |
|  |  | Noisy Pitta | *Pitta versicolor* |
|  |  | Striped Honeyeater | *Plectorhyncha lanceolata* |
|  |  | Black-Throated Finch | *Poephila cincta* |
|  |  | White-Browed Babbler | *Pomatostomus superciliosus* |
|  |  | Grey-Crowned Babbler | *Pomatostomus temporalis* |
|  |  | Eastern Whipbird | *Psophodes olivaceus* |
|  |  | Spotted Bowerbird | *Ptilonorhynchus maculatus* |
|  |  | Satin Bowerbird | *Ptilonorhynchus violaceus* |
|  |  | Paradise Riflebird | *Ptiloris paradiseus* |
|  |  | Fuscous Honeyeater | *Ptilotula fuscus* |
|  |  | Yellow-Plumed Honeyeater | *Ptilotula ornatus* |
|  |  | White-Plumed Honeyeater | *Ptilotula penicillatus* |
|  |  | Grey-Fronted Honeyeater | *Ptilotula plumulus* |
|  |  | White-Fronted Honeyeater | *Purnella albifrons* |
|  |  | Grey Fantail | *Rhipidura albiscapa* |
|  |  | Willie Wagtail | *Rhipidura leucophrys* |
|  |  | Rufous Fantail | *Rhipidura rufifrons* |
|  |  | Yellow-Throated Scrubwren | *Sericornis citreogularis* |
|  |  | White-Browed Scrubwren | *Sericornis frontalis* |
|  |  | Large-Billed Scrubwren | *Sericornis magnirostra* |
|  |  | Regent Bowerbird | *Sericulus chrysocephalus* |
|  |  | Weebill | *Smicrornis brevirostris* |
|  |  | Australasian Figbird | *Sphecotheres vieilloti* |
|  |  | Timor Figbird | *Sphecotheres viridis* |
|  |  | Beautiful Firetail | *Stagonopleura bella* |
|  |  | Diamond Firetail | *Stagonopleura guttata* |
|  |  | Yellow Honeyeater | *Stomiopera flavus* |
|  |  | Black Currawong | *Strepera fuliginosa* |
|  |  | Pied Currawong | *Strepera graculina* |
|  |  | Grey Currawong | *Strepera versicolor* |
|  |  | Apostlebird | *Struthidea cinerea* |
|  |  | Black Honeyeater | *Sugomel niger* |
|  |  | Spectacled Monarch | *Symposiachrus trivirgatus* |
|  |  | Double-Barred Finch | *Taeniopygia bichenovii* |
|  |  | Zebra Finch | *Taeniopygia guttata* |
|  |  | Pale-Yellow Robin | *Tregellasia capito* |
|  |  | Russet-Tailed Thrush | *Zoothera heinei* |
|  |  | Bassian Thrush | *Zoothera lunulata* |
|  |  | Silvereye | *Zosterops lateralis* |
|  | Pigeons and Doves | Emerald Dove | *Chalcophaps indica* |
|  |  | White-Headed Pigeon | *Columba leucomela* |
|  |  | Diamond Dove | *Geopelia cuneata* |
|  |  | Bar-Shouldered Dove | *Geopelia humeralis* |
|  |  | Peaceful Dove | *Geopelia striata* |
|  |  | Squatter Pigeon | *Geophaps scripta* |
|  |  | Wonga Pigeon | *Leucosarcia melanoleuca* |
|  |  | Topknot Pigeon | *Lopholaimus antarcticus* |
|  |  | Brown Cuckoo-Dove | *Macropygia amboinensis* |
|  |  | Crested Pigeon | *Ocyphaps lophotes* |
|  |  | Common Bronzewing | *Phaps chalcoptera* |
|  |  | Brush Bronzewing | *Phaps elegans* |
|  |  | Wompoo Fruit-Dove | *Ptilinopus magnificus* |
|  |  | Rose-Crowned Fruit-Dove | *Ptilinopus regina* |
|  |  | Superb Fruit-Dove | *Ptilinopus superbus* |
| Mammalia | Bandicoots, bettongs, and potoroos | Rufous Bettong | *Aepyprymnus rufescens* |
|  |  | Northern Brown Bandicoot | *Isoodon macrourus* |
|  |  | Southern Brown Bandicoot | *Isoodon obesulus* |
|  |  | Bilby | *Macrotis lagotis* |
|  |  | Eastern Barred Bandicoot | *Perameles gunnii* |
|  |  | Long-Nosed Bandicoot | *Perameles nasuta* |
|  |  | Long-Footed Potoroo | *Potorous longipes* |
|  |  | Long-Nosed Potoroo | *Potorous tridactylus* |
|  |  | Potorous Tridactylus Tridactylus | *Potorous tridactylus tridactylus* |
|  | Echidna | Short-Beaked Echidna | *Tachyglossus aculeatus* |
|  | Feathertail glider | Feathertail Glider | *Acrobates pygmaeus* |
|  | Flying-foxes | Eastern Tube-Nosed Bat | *Nyctimene robinsoni* |
|  |  | Black Flying-Fox | *Pteropus alecto* |
|  |  | Grey-Headed Flying-Fox | *Pteropus poliocephalus* |
|  |  | Little Red Flying-Fox | *Pteropus scapulatus* |
|  |  | Common Blossom-Bat | *Syconycteris australis* |
|  | Koala | Koala | *Phascolarctos cinereus* |
|  | Large gliders | Greater Glider | *Petauroides volans* |
|  |  | Yellow-Bellied Glider | *Petaurus australis* |
|  |  | Sugar Glider | *Petaurus breviceps* |
|  |  | Squirrel Glider | *Petaurus norfolcensis* |
|  | Large kangaroos | Western Grey Kangaroo | *Macropus fuliginosus* |
|  |  | Eastern Grey Kangaroo | *Macropus giganteus* |
|  |  | Macropus giganteus giganteus | *Macropus giganteus giganteus* |
|  |  | Common Wallaroo | *Macropus robustus* |
|  |  | Red Kangaroo | *Macropus rufus* |
|  | Marsupial dasyurid | Agile Antechinus | *Antechinus agilis* |
|  |  | Yellow-Footed Antechinus | *Antechinus flavipes* |
|  |  | Brown Antechinus | *Antechinus stuartii* |
|  |  | Dusky Antechinus | *Antechinus swainsonii* |
|  |  | Spotted-Tailed Quoll | *Dasyurus maculatus* |
|  |  | Eastern Quoll | *Dasyurus viverrinus* |
|  |  | Red-Tailed Phascogale | *Phascogale calura* |
|  |  | Brush-Tailed Phascogale | *Phascogale tapoatafa* |
|  |  | Common Planigale | *Planigale maculata* |
|  |  | Stripe-Faced Dunnart | *Sminthopsis macroura* |
|  |  | Common Dunnart | *Sminthopsis murina* |
|  | Microbats | Large-Eared Pied Bat | *Chalinolobus dwyeri* |
|  |  | Gould's Wattled Bat | *Chalinolobus gouldii* |
|  |  | Chocolate Wattled Bat | *Chalinolobus morio* |
|  |  | Eastern Forest Bat | *Eptesicus pumilus* |
|  |  | Eastern False Pipistrelle | *Falsistrellus tasmaniensis* |
|  |  | Little Bentwing-Bat | *Miniopterus australis* |
|  |  | Common Bentwing-Bat | *Miniopterus schreibersii* |
|  |  | Eastern Bentwing-Bat | *Miniopterus schreibersii oceanensis* |
|  |  | Mormopterus "Species 2" | *Mormopterus "Species 2"* |
|  |  | Mormopterus "Species 3" (Little Penis) | *Mormopterus "Species 3" (little penis)* |
|  |  | Mormopterus "Species 4" (Big Penis) | *Mormopterus "Species 4" (big penis)* |
|  |  | Beccari's Freetail-Bat | *Mormopterus beccarii* |
|  |  | Eastern Freetail-Bat | *Mormopterus norfolkensis* |
|  |  | Little Mastiff-Bat | *Mormopterus planiceps* |
|  |  | Eastern Free-Tailed Bat | *Mormopterus ridei* |
|  |  | Large-Footed Myotis | *Myotis adversus* |
|  |  | Southern Myotis | *Myotis macropus* |
|  |  | Eastern Long-Eared Bat | *Nyctophilus bifax* |
|  |  | Corben's Long-Eared Bat | *Nyctophilus corbeni* |
|  |  | Lesser Long-Eared Bat | *Nyctophilus geoffroyi* |
|  |  | Gould's Long-Eared Bat | *Nyctophilus gouldi* |
|  |  | Eastern Horseshoe-Bat | *Rhinolophus megaphyllus* |
|  |  | Yellow-Bellied Sheathtail-Bat | *Saccolaimus flaviventris* |
|  |  | Greater Broad-Nosed Bat | *Scoteanax rueppellii* |
|  |  | Inland Broad-Nosed Bat | *Scotorepens balstoni* |
|  |  | Little Broad-Nosed Bat | *Scotorepens greyii* |
|  |  | Eastern Broad-Nosed Bat | *Scotorepens orion* |
|  |  | Tadarida Australis Australis | *Tadarida australis australis* |
|  |  | White-Striped Sheathtail-Bat | *Taphozous kapalgensis* |
|  |  | Inland Forest Bat | *Vespadelus baverstocki* |
|  |  | Large Forest Bat | *Vespadelus darlingtoni* |
|  |  | Southern Forest Bat | *Vespadelus regulus* |
|  |  | Eastern Cave Bat | *Vespadelus troughtoni* |
|  |  | Little Forest Bat | *Vespadelus vulturnus* |
|  | Platypus | Platypus | *Ornithorhynchus anatinus* |
|  | Possums | Mountain Pygmy-Possum | *Burramys parvus* |
|  |  | Eastern Pygmy-Possum | *Cercartetus nanus* |
|  |  | Common Ringtail Possum | *Pseudocheirus peregrinus* |
|  |  | Short-Eared Possum | *Trichosurus caninus* |
|  |  | Mountain Brushtail Possum | *Trichosurus cunninghami* |
|  |  | Common Brushtail Possum | *Trichosurus vulpecula* |
|  | Rodents | Water-Rat | *Hydromys chrysogaster* |
|  |  | Grassland Melomys | *Melomys burtoni* |
|  |  | Fawn-Footed Melomys | *Melomys cervinipes* |
|  |  | Spinifex Hopping-Mouse | *Notomys alexis* |
|  |  | Fawn Hopping-Mouse | *Notomys cervinus* |
|  |  | Mitchell's Hopping-Mouse | *Notomys mitchellii* |
|  |  | Delicate Mouse | *Pseudomys delicatulus* |
|  |  | Desert Mouse | *Pseudomys desertor* |
|  |  | Eastern Chestnut Mouse | *Pseudomys gracilicaudatus* |
|  |  | New Holland Mouse | *Pseudomys novaehollandiae* |
|  |  | Hastings River Mouse | *Pseudomys oralis* |
|  |  | Bush Rat | *Rattus fuscipes* |
|  |  | Swamp Rat | *Rattus lutreolus* |
|  | Small macropods | Agile Wallaby | *Macropus agilis* |
|  |  | Black-Striped Wallaby | *Macropus dorsalis* |
|  |  | Parma Wallaby | *Macropus parma* |
|  |  | Whiptail Wallaby | *Macropus parryi* |
|  |  | Red-Necked Wallaby | *Macropus rufogriseus* |
|  |  | Brush-Tailed Rock-Wallaby | *Petrogale penicillata* |
|  |  | Yellow-Footed Rock-Wallaby | *Petrogale xanthopus* |
|  |  | Red-Legged Pademelon | *Thylogale stigmatica* |
|  |  | Red-Necked Pademelon | *Thylogale thetis* |
|  |  | Swamp Wallaby | *Wallabia bicolor* |
|  | Wombat | Northern Hairy-Nosed Wombat | *Lasiorhinus krefftii* |
|  |  | Common Wombat | *Vombatus ursinus* |
| Reptilia | Bearded dragons and blue tongues | Land Mullet | *Bellatorias major* |
|  |  | Bearded Dragon | *Pogona barbata* |
|  |  | Downs Bearded Dragon | *Pogona henrylawsoni* |
|  |  | Central Bearded Dragon | *Pogona vitticeps* |
|  |  | Blotched Blue-Tongue | *Tiliqua nigrolutea* |
|  |  | Western Blue-Tongued Lizard | *Tiliqua occipitalis* |
|  |  | Shingle-Back | *Tiliqua rugosa* |
|  |  | Eastern Blue-Tongue | *Tiliqua scincoides* |
|  | Freshwater turtle | Broad-Shelled Turtle | *Chelodina expansa* |
|  |  | Eastern Snake-Necked Turtle | *Chelodina longicollis* |
|  |  | Mary River Tortoise | *Elusor macrurus* |
|  |  | Macquarie Turtle | *Emydura macquarii* |
|  |  | Macleay River Turtle | *Emydura macquarii dharra* |
|  |  | Hunter River Turtle | *Emydura macquarii gunabarra* |
|  |  | Krefft's River Turtle | *Emydura macquarii krefftii* |
|  |  | Macquarie River Turtle | *Emydura macquarii macquarii* |
|  |  | Fraser Island Turtle | *Emydura macquarii nigra* |
|  |  | Brisbane River Turtle | *Emydura macquarii signata* |
|  |  | George's Turtle | *Wollumbinia georgesi* |
|  |  | Saw-Shelled Turtle | *Wollumbinia latisternum* |
|  | Geckoes | Lesueur's Velvet Gecko | *Amalosia lesueurii* |
|  |  | Marbled Gecko | *Christinus marmoratus* |
|  |  | Fat-Tailed Gecko | *Diplodactylus platyurus* |
|  |  | Tessellated Gecko | *Diplodactylus tessellatus* |
|  |  | Bynoe's Gecko | *Heteronotia binoei* |
|  |  | Robust Velvet Gecko | *Nebulifera robusta* |
|  |  | Smooth Knob-Tail | *Nephrurus laevissimus* |
|  |  | Northern Velvet Gecko | *Oedura castelnaui* |
|  |  | Marbled Velvet Gecko | *Oedura marmorata* |
|  |  | Northern Leaf-Tailed Gecko | *Phyllurus cornutus* |
|  |  | Broad-Tailed Gecko | *Phyllurus platurus* |
|  |  | Southern Leaf-Tailed Gecko | *Saltuarius swaini* |
|  |  | Eastern Spiny-Tailed Gecko | *Strophurus williamsi* |
|  |  | Border Thick-Tailed Gecko | *Uvidicolus sphyrurus* |
|  | Large reptiles and monitors | Frilled Lizard | *Chlamydosaurus kingii* |
|  |  | Eastern Water Dragon | *Intellagama lesueurii* |
|  |  | Gould's Goanna | *Varanus gouldii* |
|  |  | Rosenberg's Goanna | *Varanus rosenbergi* |
|  |  | Storr's Monitor | *Varanus storri* |
|  |  | Varanus tristis orientalis | *Varanus tristis orientalis* |
|  |  | Lace Monitor | *Varanus varius* |
|  | Small skinks and lizards | Jacky Lizard | *Amphibolurus muricatus* |
|  |  | Two-Clawed Worm-Skink | *Anomalopus lentiginosus* |
|  |  | Punctate Worm-Skink | *Anomalopus swansoni* |
|  |  | Three-Clawed Worm-Skink | *Anomalopus verreauxii* |
|  |  | Legless Lizard | *Aprasia aurita* |
|  |  | Pink-Tailed Legless Lizard | *Aprasia parapulchella* |
|  |  | Major Skink | *Bellatorias frerei* |
|  |  | Robust Rainbow-Skink | *Carlia schmeltzii* |
|  |  | Three-Toed Snake-Tooth Skink | *Coeranoscincus reticulatus* |
|  |  | Tawny Crevice-Dragon | *Ctenophorus decresii* |
|  |  | Central Netted Dragon | *Ctenophorus nuchalis* |
|  |  | Copper-Tailed Skink | *Ctenotus taeniolatus* |
|  |  | Tasmanian She-Oak Skink | *Cyclodomorphus casuarinae* |
|  |  | Pink-Tongued Lizard | *Cyclodomorphus gerrardii* |
|  |  | Patternless Delma | *Delma inornata* |
|  |  | Tommy Roundhead | *Diporiphora australis* |
|  |  | Cunningham's Skink | *Egernia cunninghami* |
|  |  | Black Rock Skink | *Egernia saxatilis* |
|  |  | Gidgee Skink | *Egernia stokesii* |
|  |  | Tree Skink | *Egernia striolata* |
|  |  | Blue Mountains Water Skink | *Eulamprus leuraensis* |
|  |  | Eastern Water-Skink | *Eulamprus quoyii* |
|  |  | Barred-Sided Skink | *Eulamprus tenuis* |
|  |  | Dark-Flecked Garden Sunskink | *Lampropholis delicata* |
|  |  | Pale-Flecked Garden Sunskink | *Lampropholis guichenoti* |
|  |  | Burton's Snake-Lizard | *Lialis burtonis* |
|  |  | Common Dwarf Skink | *Menetia greyii* |
|  |  | Fire-Tailed Skink | *Morethia taeniopleura* |
|  |  | Common Scaly-Foot | *Pygopus lepidopodus* |
|  |  | Western Hooded Scaly-Foot | *Pygopus nigriceps* |
|  |  | Mountain Dragon | *Rankinia diemensis* |
|  |  | Three-Toed Skink | *Saiphos equalis* |
|  |  | Tiliqua Scincoides Scincoides | *Tiliqua scincoides scincoides* |
|  | Snakes - others | Common Death Adder | *Acanthophis antarcticus* |
|  |  | Small-Headed Blind Snake | *Anilios affinis* |
|  |  | Southern Blind Snake | *Anilios australis* |
|  |  | Prong-Snouted Blind Snake | *Anilios bituberculatus* |
|  |  | Robust Blind Snake | *Anilios ligatus* |
|  |  | Small-Eyed Blind Snake | *Anilios micromma* |
|  |  | Blackish Blind Snake | *Anilios nigrescens* |
|  |  | Proximus Blind Snake | *Anilios proximus* |
|  |  | Brown-Snouted Blind Snake | *Anilios wiedii* |
|  |  | Woma | *Aspidites ramsayi* |
|  |  | Highland Copperhead | *Austrelaps ramsayi* |
|  |  | Brown Tree Snake | *Boiga irregularis* |
|  |  | Coral Snake | *Brachyurophis australis* |
|  |  | White-Crowned Snake | *Cacophis harriettae* |
|  |  | Southern Dwarf Crowned Snake | *Cacophis krefftii* |
|  |  | Golden-Crowned Snake | *Cacophis squamulosus* |
|  |  | Eastern Small-Eyed Snake | *Cryptophis nigrescens* |
|  |  | Central Military Dragon | *Ctenophorus isolepis* |
|  |  | Yellow-Faced Whip Snake | *Demansia psammophis* |
|  |  | Common Tree Snake | *Dendrelaphis punctulatus* |
|  |  | White-Lipped Snake | *Drysdalia coronoides* |
|  |  | Masters' Snake | *Drysdalia mastersii* |
|  |  | Mustard-Bellied Snake | *Drysdalia rhodogaster* |
|  |  | Red-Naped Snake | *Furina diadema* |
|  |  | Black-Bellied Swamp Snake | *Hemiaspis signata* |
|  |  | Broad-Headed Snake | *Hoplocephalus bungaroides* |
|  |  | Stephens' Banded Snake | *Hoplocephalus stephensii* |
|  |  | Tiger Snake | *Notechis scutatus* |
|  |  | Eastern Or Mainland Tiger Snake | *Notechis scutatus scutatus* |
|  |  | Taipan | *Oxyuranus scutellatus* |
|  |  | Dwyer's Snake | *Parasuta dwyeri* |
|  |  | King Brown Snake | *Pseudechis australis* |
|  |  | Spotted Black Snake | *Pseudechis guttatus* |
|  |  | Red-Bellied Black Snake | *Pseudechis porphyriacus* |
|  |  | Ringed Brown Snake | *Pseudonaja modesta* |
|  |  | Western Brown Snake | *Pseudonaja nuchalis* |
|  |  | Eastern Brown Snake | *Pseudonaja textilis* |
|  |  | Narrow-Banded Snake | *Simoselaps fasciolatus* |
|  |  | Little Whip Snake | *Suta flagellum* |
|  |  | Rough-Scaled Snake | *Tropidechis carinatus* |
|  |  | Freshwater Snake | *Tropidonophis mairii* |
|  |  | Bandy-Bandy | *Vermicella annulata* |
|  | Snakes - Pythons | Children's Python | *Antaresia childreni* |
|  |  | Children's/Eastern Small-Blotched Python Hybrid | *Antaresia childreni x maculosa* |
|  |  | Children's/Stimson's Python Hybrid | *Antaresia childreni x stimsoni* |
|  |  | Spotted Python | *Antaresia maculosa* |
|  |  | Stimson's Python | *Antaresia stimsoni* |
|  |  | Amethyst Python | *Morelia amethistina* |
|  |  | Centralian Carpet Python | *Morelia bredli* |
|  |  | Rough-Scaled Python | *Morelia carinata* |
|  |  | Carpet & Diamond Pythons | *Morelia spilota* |
|  |  | Jungle Carpet Python | *Morelia spilota cheyni* |
|  |  | Eastern Carpet Python | *Morelia spilota mcdowelli* |
|  |  | Coastal/Centralian Carpet Python Hybrid | *Morelia spilota mcdowelli x bredli* |
|  |  | Murray/Darling Carpet Python | *Morelia spilota metcalfei* |
|  |  | Diamond Python | *Morelia spilota spilota* |
|  |  | Diamond/Jungle Python Hybrid | *Morelia spilota spilota x cheynei* |
|  |  | Carpet/Diamond Python | *Morelia spilota spilota x mcdowelli* |
|  |  | Top End Carpet Python | *Morelia spilota variegata* |
|  |  | Green Python | *Morelia viridis* |
