# Supplementary figures and images for: Trends in wildlife rehabilitation rescues and animal fate across a six-year period in New South Wales, Australia
Source: PLoS One. 2021 Sep 10;16(9):e0257209. doi: 10.1371/journal.pone.0257209 (PMC8432793; doi:10.1371/journal.pone.0257209)

**S4 File: Species accumulation curves for birds, mammals, and reptiles**

**Birds:**


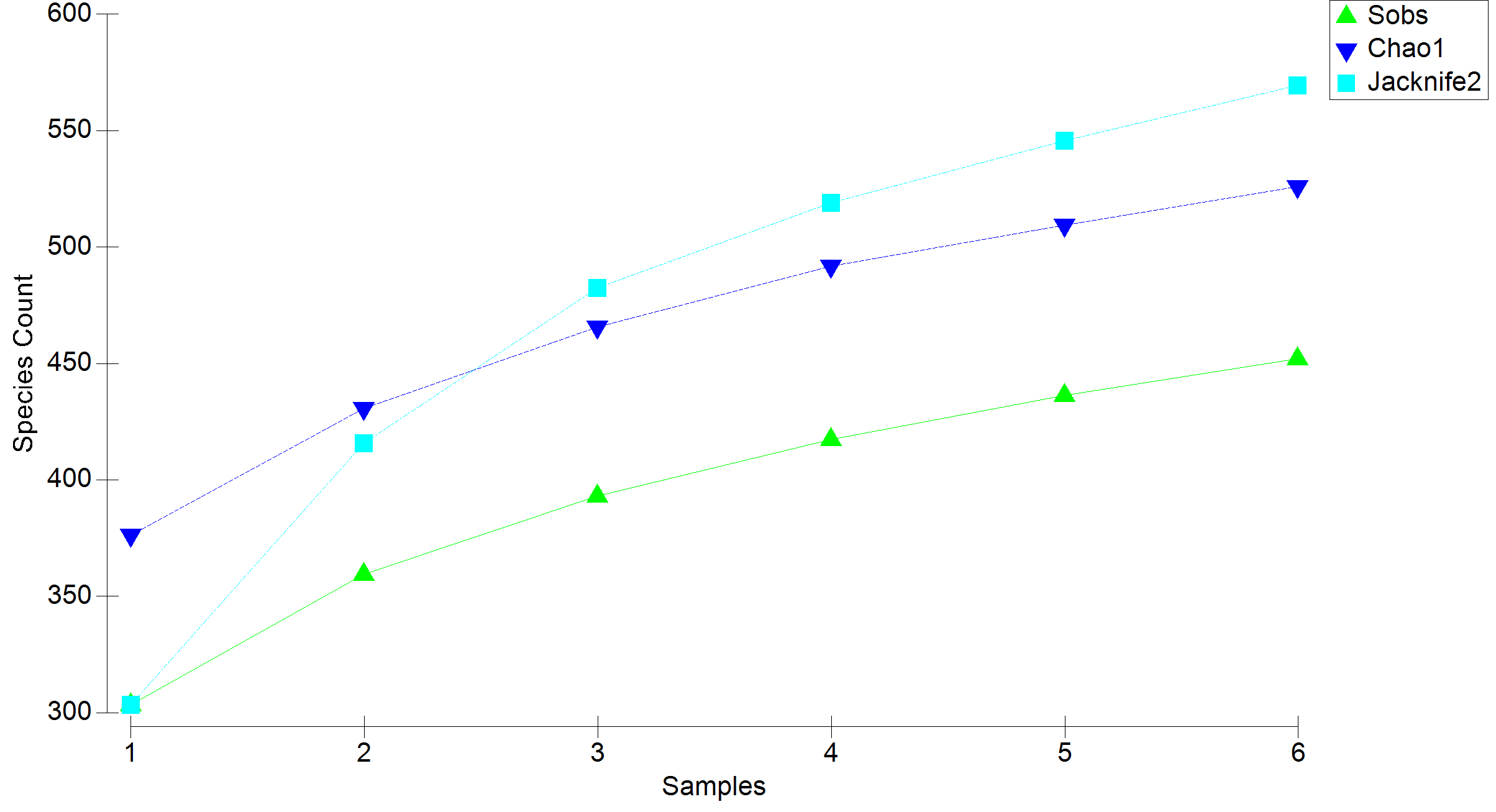


**Mammals:**

**
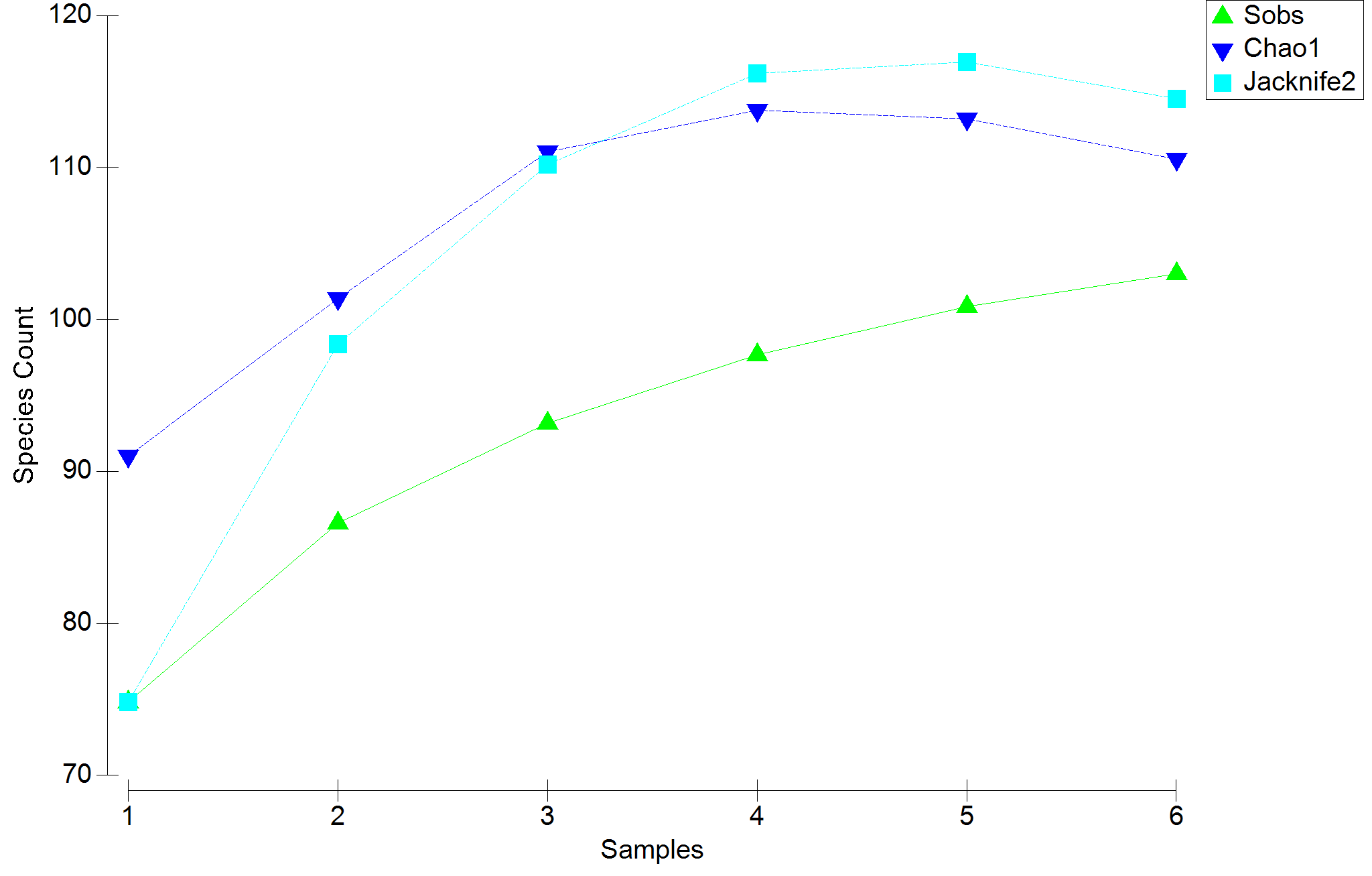
**

**Reptiles:**

**
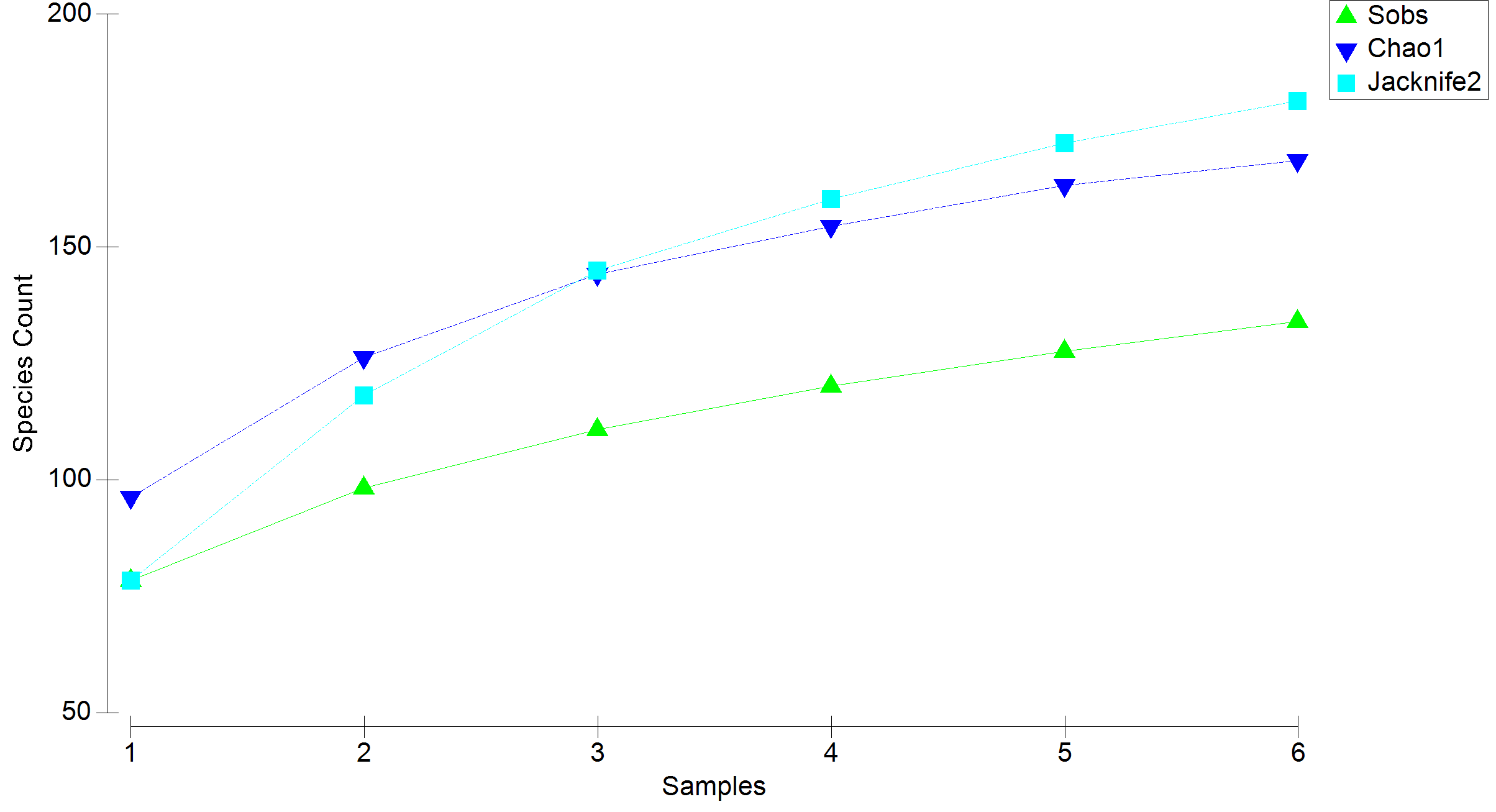
**

Supplement: S5 File — (DOCX) [file pone.0257209.s005.docx]
